# *In-silico* identification and characterization of *O-methyltransferase* gene family in peanut (*Arachis hypogaea* L.) reveals their putative roles in development and stress tolerance

**DOI:** 10.3389/fpls.2023.1145624

**Published:** 2023-03-31

**Authors:** Tiecheng Cai, Yasir Sharif, Yuhui Zhuang, Qiang Yang, Xiangyu Chen, Kun Chen, Yuting Chen, Meijia Gao, Hao Dang, Yijing Pan, Ali Raza, Chong Zhang, Hua Chen, Weijian Zhuang

**Affiliations:** ^1^ Center of Legume Plant Genetics and System Biology, College of Agronomy, Fujian Agriculture and Forestry University (FAFU), Fuzhou, Fujian, China; ^2^ College of Life Science, Fujian Agriculture and Forestry University, Fuzhou, Fujian, China; ^3^ Crops Research Institute, Fujian Academy of Agricultural Science, Fuzhou, Fujian, China; ^4^ College of Plant Protection, Fujian Agriculture and Forestry University, Fuzhou, Fujian, China

**Keywords:** bioinformatics, environmental stress, functional annotation, gene duplication, micro-RNAs, peanut genomics, phylogenetic tree

## Abstract

Cultivated peanut (*Arachis hypogaea*) is a leading protein and oil-providing crop and food source in many countries. At the same time, it is affected by a number of biotic and abiotic stresses. O-methyltransferases (*OMTs*) play important roles in secondary metabolism, biotic and abiotic stress tolerance. However, the *OMT* genes have not been comprehensively analyzed in peanut. In this study, we performed a genome-wide investigation of *A. hypogaea OMT* genes (*AhOMTs*). Gene structure, motifs distribution, phylogenetic history, genome collinearity and duplication of *AhOMTs* were studied in detail. Promoter *cis*-elements, protein-protein interactions, and micro-RNAs targeting *AhOMTs* were also predicted. We also comprehensively studied their expression in different tissues and under different stresses. We identified 116 *OMT* genes in the genome of cultivated peanut. Phylogenetically, *AhOMTs* were divided into three groups. Tandem and segmental duplication events played a role in the evolution of *AhOMTs*, and purifying selection pressure drove the duplication process. *AhOMT* promoters were enriched in several key *cis*-elements involved in growth and development, hormones, light, and defense-related activities. Micro-RNAs from 12 different families targeted 35 *AhOMTs*. GO enrichment analysis indicated that *AhOMTs* are highly enriched in transferase and catalytic activities, cellular metabolic and biosynthesis processes. Transcriptome datasets revealed that *AhOMTs* possessed varying expression levels in different tissues and under hormones, water, and temperature stress. Expression profiling based on qRT-PCR results also supported the transcriptome results. This study provides the theoretical basis for further work on the biological roles of *AhOMT* genes for developmental and stress responses.

## Introduction

In *Arabidopsis thaliana*, *O-methyltransferases* (*OMTs*) are heterogeneous enzymes involved in the flavonoid and lignin production pathways (Guo et al., 2001). There are three classes of plant *methyltransferases*: *C*- *methyltransferases*, *N*- *methyltransferases*, and *O-methyltransferases* (Roje, 2006). In plants, *OMTs* assist the transfer of the methyl group of *S-adenosyl-L-methionine* (SAM) to the hydroxyl group of numerous organic chemical compounds, ultimately synthesizing the methyl ether variants of these substances (Struck et al., 2012). Based on the molecular weight and bivalent ion dependence, OMTs are divided into *Caffeoyl-CoA OMT* (*CCoAOMT*) and *Caffeic acid OMT* (*COMT*). *COMTs* are the main representative of type I, and *CCoAOMTs* are of type II (Davin and Lewis, 1992). Depending upon the resemblance in sequence and protein motifs, *OMT* genes are further classified into two separate categories: PL-OMT I and PL-OMT II (*CCoAOMT* and *COMT*, respectively) (Joshi and Chiang, 1998). *COMT*-type proteins bind to a variety of substrates, including caffeoyl CoA ester, caffeic acid, chalcones, myoinositol, scoulerine, 5-hydroxyferuloylester, and 5-hydroxyferulic acid (Ye et al., 1994; Roje, 2006). *CCoAOMT*-type enzymes use a pair of substrates, caffeoyl CoA and 5-hydroxyferuloyl CoA, to function (Davin and Lewis, 1992). *COMT* and *CCoAOMT* both mediate the lignin biosynthesis process. The *CCoAOMT* enzyme catalyzes an early step in the pathway by converting *caffeoyl CoA* to *feruloyl CoA* (Dudareva and Pichersky, 2008), despite the fact that sinapyl alcohol, a key component of S-type lignin, is mostly biosynthesized by *COMT* proteins at the end of the biosynthetic pathway (Ye et al., 1994; Buer et al., 2010).

Lignin is the second most prevalent biopolymer on the planet and is an essential element of cell walls in certain higher plants (Ralph et al., 2004). It offers mechanical strength to plants and assists water movement throughout whole plant tissues (Liu et al., 2018), and also an excellent barricade for pathogens, fungi, and insects (Peng et al., 2014), so it helps to improve plant response toward environmental calamities (Moura et al., 2010). To understand their significance, *OMT* genes have been extensively studied in various plants, such as *Arabidopsis* and rice (Hamberger et al., 2007), citrus and sorghum (Liu et al., 2016b; Rakoczy et al., 2018), switchgrass and dove tree (LIU et al., 2016a), tea plant (Lin et al., 2021) etc. Concerning wheat, Nguyen and his team analyzed the expression profiles of lignin biosynthesis-related genes, including a number of *CCoAOMTs*, to determine the likely mechanisms behind their expression patterns. They discovered that lignin content was directly linked with lodging resistance, tolerance to various biotic and abiotic stresses, and quality of feedstock biomass (Nguyen et al., 2016). *TaCCoAOMT1* regulates lignin biosynthesis (Ma and Luo, 2015); previously, this gene has been reported as a key stem cell growth regulator (Bi et al., 2011). Due to their significant roles in secondary metabolism, intensive work has been done on *OMT* genes throughout the years (Bout and Vermerris, 2003; Goujon et al., 2003; Kota et al., 2004; Li et al., 2006; Lin et al., 2006; Yoshihara et al., 2008; Ma, 2009; Zhou et al., 2009). A detailed evaluation of the *OMT* genes in peanut has yet to be performed, despite the fact that the genes’ well-established role offers a good foundation for our research.

Therefore, *OMT* genes were studied at a genome-wide scale in *A. hypogaea* and its wild progenitors. One hundred and sixteen *OMT* genes were found in the cultivated peanut genome. Further, we looked into the evolutionary connections of these *AhOMT* genes, their conserved domains and motifs, gene structure, and genomic position. We likewise investigated the *AhOMT* promoters; similarly, expression in different organs under various stress conditions was investigated as well. This study will provide a base for further research on individual genes in peanut and will aid in exploring the biological roles of the *OMT* genes.

## Materials and methods

### Identification and characterization of *OMT* genes in *A. hypogaea*

*OMT* genes in the genome of *A. hypogaea* were comprehensively searched. The protein sequences of *AtOMTs* were acquired from the TAIR database (https://www.arabidopsis.org/) (Lamesch et al., 2012) and soybean *OMTs* from Legume Information System (https://legumeinfo.org/) (Gonzales et al., 2005). *A. ipaensis* and *A. duranensis OMT* sequences were obtained from the PeanutBase database (https://www.peanutbase.org/home) (Bertioli et al., 2016). The sequences of whole-genome proteins of *A. hypogaea* were obtained from the Peanut Genome Resource database (PGR) (http://peanutgr.fafu.edu.cn/) (Zhuang et al., 2019). The protein sequences of *OMTs* from *A. duranensis*, *A. ipaensis*, *A. thaliana*, and *G. max* were used to search the *AhOMTs* by BLASTP search with TBtools software (Chen et al., 2020). Further, the HMM search method was also used to search the *OMT* proteins from *A. hypogaea* genome. The Pfam database was searched to obtain the HMM files for the *OMT* family (PF08100 and PF00891) (http://pfam.xfam.org/). The identified proteins were scanned at NCBI and Pfam databases to verify the *OMT* domain. ProtParam tool (http://web.expasy.org/protparam/) determined the physicochemical characteristics of *AhOMTs* (Gasteiger et al., 2005). The subcellular localizations of *AhOMT* proteins in different cell organelles were predicted by the CELLO version v2.5 (http://cello.life.nctu.edu.tw/) (Yu et al., 2006). General Feature Format (GFF3) files were used to view the exon-intron distribution pattern of *AhOMTs* through TBtools software. Conserved motifs of *AhOMT* proteins were determined by the MEME database (https://meme-db.org/motifs/) (Bailey et al., 2015).

### Phylogenetic and gene duplication analysis of *AhOMTs*

A phylogenetic tree comprising *A. ipaensis, A. duranensis, G. max*, *A. hypogaea*, and *A. thaliana* proteins was constructed to investigate their phylogenetic connections. Protein sequences were subjected to multiple sequence alignment by MUSCLE method with the help of MEGAX software (https://megasoftware.net/home) (Kumar et al., 2018). A neighbor-joining tree was generated through 1,000 bootstraps with the poisson model. MCScanX was run to identify the duplicated genes. The KaKs Calculator 2.0 program with the MYN approach was used to determine the rates of synonymous and nonsynonymous substitution (Wang et al., 2010). T = ks/2r was used to compute the divergence time with the neutral substitution coefficient r=8.12×10^-9^ (Bertioli et al., 2016).

### Analysis of *AhOMT* promoters and miRNAs prediction

Promoter sequences up to 2 kb were used to find different binding cites and cis-elements through the PlantCARE database (http://bioinformatics.psb.ugent.be/webtools/plantcare/html/) (Lescot et al., 2002). Coding sequences of *AhOMTs* were used to identify putative miRNAs targeting the *AhOMT* genes through the psRNATarget database (https://www.zhaolab.org/psRNATarget/home) (Dai et al., 2018).

### Genome collinearity and orthologous gene clusters

Comparative synteny was analyzed to examine evolutionary genome conservations between three peanut species and *Arabidopsis*. The genome and GFF3 files of all these species were subjected to McScanX in TBtools software, and the resulting files were used for multiple synteny analysis. The orthologous *OMT* proteins were identified in *A. hypogea*, *A. duranensis*, *A. ipaensis*, and *A. thaliana* through OthroVenn2 (https://orthovenn2.bioinfotoolkits.net/home) (Xu et al., 2019). Protein sequences of *Arabidopsis*, soybean, and three peanut species were used to identify orthologous genes. The peanut species were assessed individually with each other and with *Arabidopsis* and soybean to identify orthologous gene clusters.

### Functional annotation and prediction of protein-protein interactions

For functional annotation prediction (GO and KEGG), *AhOMT* proteins were scanned at the EggNOG database (http://eggnog-mapper.embl.de/) (Huerta-Cepas et al., 2019). Enrichment analyses were executed in TBtools software from predicted GO and KEGG annotations.

Protein-protein interactions were predicted based on studied *AtOMTs*. STRING 11.5 tool (https://www.string-db.org/cgi/) (Szklarczyk et al., 2019) was used to construct the interaction network between peanut and *Arabidopsis OMTs*. The top 10 interactions were predicted with a medium threshold level (0.4). MCL clustering with inflation parameter 10 was used, and dotted lines were used between cluster edges.

### Expression profiling of *AhOMT* Gsenes

Transcriptome expression data were accessed to view the expression levels of *AhOMTs* in various organs, phytohormones, water, and temperature treatments. Transcriptome expression data for different tissues (leaf, stem, stem tip, fluorescence, root, root and stem, root tip, root nodule, gynophore/peg, pericarp, testa, cotyledons, and embryo), hormones (ABA, SA, brassinolide, paclobutrazol, ethephon, and ddH_2_O as control), water (drought and normal irrigation) and temperature treatments (low temperature and room temperature) were accessed from the PGR database. The log2 normalization Fragments per kilobase million (FPKM) of *AhOMTs* were used to construct the heatmaps.

### Stress treatments and qRT-PCR analysis

Seedlings of peanut cultivar Minhua 6 (M-6) were grown in the greenhouse for stress treatments. Four-leaf old M-6 plants were subjected to abscisic acid stress (ABA 10 μg/mL) and low temperature (4°C). Samples were collected before treatment (0h, CK) for both ABA and low temperature and 3, 6, 9, and 12 hours after treatment. RNA was extracted by the CTAB method with some modifications (Sharif et al., 2022). cDNA was synthesized by Evo M-MLV RT Kit (Accurate Biotechnology, Hunan, Co., Ltd. China) following the manufacturer’s protocol. qRT-PCR was performed following our previous study (Sharif et al., 2022), while peanut *Actin* gene was used as the internal control. Data were analyzed by the 2^-ΔΔC_T_^ method (Livak and Schmittgen, 2001). Expression levels at different time points were subjected to analysis of variance (ANOVA) and LSD test at α=0.05. Primers used for qRT-PCR are given in **Supplementary Table 1**.

## Results

### Identification and characterization of *OMT* genes in *A. hypogaea*

BLASTP and HMM searches were performed to find out the *AhOMT* family genes. Twenty-four genes were found in *Arabidopsis*, 55 in *G. max*, 58 in *A. duranensis*, and 68 in *A. ipaensis* through a comprehensive search in their respective genome databases. BLASTP search using these proteins and HMM search identified 116 *OMT* genes in the *A. hypogaea* genome. **Table 1** shows the details of all 116 *AhOMT* genes. Briefly, *AhOMT* genes varied in size, ranging from 57aa (*AhOMT84* and *AhOMT110*) to 449aa (*AhOMT63*). The same genes possessed the shortest and longest CDS lengths: (*AhOMT84*, *AhOMT110*) with 174bp and *AhOMT63* with 1350bp. The physicochemical properties of these genes also varied accordingly. The molecular weights were from 6.537 kDa (*AhOMT84* and *AhOMT110*) to 502.99 kDa (*AhOMT63*), and theoretical isoelectric points varied from 4.5 (*AhOMT84*, *AhOMT110*) to 9.06 (*AhOMT108*). The differences in isoelectric point (pI) and molecular weights (MW) are attributable to post-translational modifications and a high concentration of basic amino acids.

**Table 1:** Identified OMT genes in *Arachis hypogaea* genome and their physicochemical properties.

mRNA ID	Renamed	Genomic position	Protein (aa)	CDS (bp)	Exons	MW (Da)	pI	Subcellular localization
AH00G01370.1	*AhOMT1*	Chr00, 1743491…1746458, +	367	1104	2	41630.02	5.82	Cytoplasmic
AH01G10670.1	*AhOMT2*	Chr01, 14636118…14641001, -	252	759	4	28709.05	5.85	Cytoplasmic/Nuclear
AH01G10690.1	*AhOMT3*	Chr01, 14941212…14944770, +	243	732	4	27654.91	5.29	Cytoplasmic
AH01G14360.1	*AhOMT4*	Chr01, 35441302…35442524, -	367	1104	1	40114.28	5.02	Cytoplasmic
AH02G04460.1	*AhOMT5*	Chr02, 5569873…5573618, -	386	1161	4	42470.84	5.44	Cytoplasmic
AH02G04490.1	*AhOMT6*	Chr02, 5589981…5593675, -	385	1158	4	42375.64	5.44	Cytoplasmic
AH02G12590.1	*AhOMT7*	Chr02, 32788056…32790140, -	136	411	3	15838.53	8.7	Extracellular
AH02G16370.1	*AhOMT8*	Chr02, 64332037…64333326, -	229	690	3	25721.55	5.21	Cytoplasmic
AH03G14330.1	*AhOMT9*	Chr03, 20628097…20629889, -	353	1062	2	39373.4	5.62	Cytoplasmic
AH03G37380.1	*AhOMT10*	Chr03, 129298299…129299491, -	365	1098	1	41012.78	5.73	Cytoplasmic
AH05G20880.1	*AhOMT11*	Chr05, 86349327…86352613, +	370	1113	2	41370.39	5.34	Cytoplasmic
AH05G25050.1	*AhOMT12*	Chr05, 93242846…93246353, +	238	717	5	26615.79	4.88	Cytoplasmic
AH05G37230.1	*AhOMT13*	Chr05, 113557257…113558753, +	344	1035	2	38242.97	5.78	Cytoplasmic
AH07G11630.1	*AhOMT14*	Chr07, 16284062…16284768, +	121	366	2	13465.62	4.95	PlasmaMembrane
AH07G11650.1	*AhOMT15*	Chr07, 16301351…16313771, -	366	1101	4	40534.71	5.24	Cytoplasmic
AH07G11670.1	*AhOMT16*	Chr07, 16369775…16372965, +	200	603	3	22486.09	5.96	Cytoplasmic
AH07G11680.1	*AhOMT17*	Chr07, 16456841…16459427, +	367	1104	4	40435.58	5.91	Cytoplasmic
AH07G11850.1	*AhOMT18*	Chr07, 16692680…16694596, -	365	1098	2	41236.48	5.37	Cytoplasmic
AH07G12680.1	*AhOMT19*	Chr07, 18937780…18939658, +	280	843	2	31266.38	6.14	Cytoplasmic/Mitochondrial
AH07G12700.1	*AhOMT20*	Chr07, 19043156…19045086, +	360	1083	2	40509.67	5.2	Cytoplasmic
AH07G12730.1	*AhOMT21*	Chr07, 19167296…19169130, +	359	1080	2	40469.82	5.23	Cytoplasmic
AH07G12760.1	*AhOMT22*	Chr07, 19308477…19309711, +	192	579	2	21730.12	7.84	Nuclear
AH07G12770.1	*AhOMT23*	Chr07, 19319340…19319576, +	78	237	1	8935.4	4.86	Cytoplasmic/Nuclear
AH07G12810.1	*AhOMT24*	Chr07, 19440775…19449768, -	365	1098	2	40817.08	5.55	Cytoplasmic
AH07G12840.1	*AhOMT25*	Chr07, 19516967…19518887, -	367	1104	2	41222.72	5.9	Cytoplasmic
AH07G12900.1	*AhOMT26*	Chr07, 19764268…19768113, -	428	1287	3	47972.23	5.98	Cytoplasmic
AH07G23120.1	*AhOMT27*	Chr07, 76086770…76090019, -	310	933	5	34361.73	8.64	Chloroplast
AH07G23750.1	*AhOMT28*	Chr07, 78005200…78008162, -	237	714	4	26289.16	7.06	Mitochondrial/Cytoplasmic
AH08G27130.1	*AhOMT29*	Chr08, 47394140…47396474, -	377	1134	2	42882.73	6.52	Cytoplasmic
AH09G01720.1	*AhOMT30*	Chr09, 2012805…2015264, -	367	1104	3	41450.75	5.57	Cytoplasmic
AH09G34670.1	*AhOMT31*	Chr09, 120176827…120179854, +	205	618	6	22778.35	4.72	PlasmaMembrane/Cytoplasmic
AH10G02290.1	*AhOMT32*	Chr10, 1961715…1962889, +	361	1086	1	40872.35	5.6	Cytoplasmic
AH10G15020.1	*AhOMT33*	Chr10, 54238111…54240140, +	248	747	3	27918	5.54	Cytoplasmic
AH10G16660.1	*AhOMT34*	Chr10, 74583515…74589326, +	365	1098	4	40023.61	5.67	Cytoplasmic
AH10G18790.1	*AhOMT35*	Chr10, 88278618…88281277, -	360	1083	4	40453.18	5.59	Cytoplasmic
AH10G18800.1	*AhOMT36*	Chr10, 88297763…88305007, -	366	1101	4	40511.62	6.09	Cytoplasmic/Chloroplast/Mitochondrial
AH10G32230.1	*AhOMT37*	Chr10, 114148160…114150510, -	366	1101	2	40547.15	5.92	Cytoplasmic
AH10G32240.1	*AhOMT38*	Chr10, 114152916…114155305, -	365	1098	3	40542.23	5.66	Cytoplasmic
AH10G32250.1	*AhOMT39*	Chr10, 114157814…114159860, -	361	1086	2	40403.42	5.97	Cytoplasmic
AH11G10250.1	*AhOMT40*	Chr11, 18606924…18610941, -	252	759	4	28733.08	5.72	Cytoplasmic/Nuclear
AH11G10290.1	*AhOMT41*	Chr11, 18873867…18877209, +	243	732	4	27629.84	5.19	Cytoplasmic
AH11G14590.1	*AhOMT42*	Chr11, 41545738…41546978, +	368	1107	1	40200.38	5.07	Cytoplasmic
AH12G04920.1	*AhOMT43*	Chr12, 6605031…6609257, -	386	1161	5	42419.84	5.45	Cytoplasmic
AH12G04930.1	*AhOMT44*	Chr12, 6637640…6658360, -	385	1158	4	42338.6	5.5	Cytoplasmic
AH12G19430.1	*AhOMT45*	Chr12, 87354252…87355625, -	229	690	3	25723.56	5.21	Cytoplasmic
AH13G16990.1	*AhOMT46*	Chr13, 21399995…21403296, -	360	1083	3	40162.33	5.62	Cytoplasmic
AH13G18140.1	*AhOMT47*	Chr13, 23575873…23692960, -	362	1089	2	40791.14	6.01	Cytoplasmic/Mitochondrial
AH13G18150.1	*AhOMT48*	Chr13, 23582222…23583001, -	259	780	1	28803.22	5.71	Cytoplasmic
AH13G18180.1	*AhOMT49*	Chr13, 23740970…23743323, -	362	1089	2	40836.95	5.62	Cytoplasmic
AH13G40550.1	*AhOMT50*	Chr13, 130194454…130195643, -	365	1098	1	41041.81	5.66	Cytoplasmic
AH13G54850.1	*AhOMT51*	Chr13, 146141983…146144481, +	361	1086	4	40440.53	6.12	Cytoplasmic
AH13G54860.1	*AhOMT52*	Chr13, 146149238…146151105, +	367	1104	2	40773.52	5.81	Cytoplasmic
AH13G54880.1	*AhOMT53*	Chr13, 146162045…146163928, +	370	1113	2	41220.02	6.01	Cytoplasmic
AH13G54900.1	*AhOMT54*	Chr13, 146172622…146175803, +	367	1104	3	40778.33	5.67	Cytoplasmic
AH13G54910.1	*AhOMT55*	Chr13, 146190691…146192373, +	367	1104	3	40907.59	5.92	Cytoplasmic
AH14G35680.1	*AhOMT56*	Chr14, 125806126…125811662, +	369	1110	3	41468.64	5.61	Cytoplasmic
AH14G35740.1	*AhOMT57*	Chr14, 125872352…125874795, +	369	1110	3	41683.11	5.79	Cytoplasmic
AH14G35970.1	*AhOMT58*	Chr14, 126140382…126143911, -	367	1104	3	42115.6	5.55	Cytoplasmic
AH14G35990.1	*AhOMT59*	Chr14, 126193089…126196501, -	367	1104	3	42007.48	5.4	Cytoplasmic/PlasmaMembrane
AH14G36310.1	*AhOMT60*	Chr14, 126627551…126635090, -	293	882	3	32983.34	5.69	Cytoplasmic
AH14G36320.1	*AhOMT61*	Chr14, 126649959…126651981, +	363	1092	2	41410.16	5.6	Cytoplasmic
AH14G36340.1	*AhOMT62*	Chr14, 126673606…126675256, -	266	801	2	29884.76	5.21	Cytoplasmic/PlasmaMembrane/Chloroplast
AH14G36350.1	*AhOMT63*	Chr14, 126701052…126704074, -	449	1350	4	50299.16	5.3	Cytoplasmic
AH14G37140.1	*AhOMT64*	Chr14, 127461985…127463907, -	357	1074	2	40448.73	5.97	Cytoplasmic
AH14G37150.1	*AhOMT65*	Chr14, 127470799…127472796, -	362	1089	2	40849.08	5.21	Cytoplasmic
AH14G37180.1	*AhOMT66*	Chr14, 127485031…127487122, -	362	1089	2	40883.95	5.03	Cytoplasmic
AH14G37190.1	*AhOMT67*	Chr14, 127510636…127512447, -	362	1089	2	40819.03	5.04	Cytoplasmic
AH14G37200.1	*AhOMT68*	Chr14, 127525946…127528357, +	359	1080	3	40298.99	6.38	Cytoplasmic
AH14G39080.1	*AhOMT69*	Chr14, 129224323…129226281, +	212	639	3	24003.78	6.51	Cytoplasmic
AH14G39130.1	*AhOMT70*	Chr14, 129291044…129293057, -	265	798	2	29330.54	6.07	Cytoplasmic
AH14G39140.1	*AhOMT71*	Chr14, 129294783…129302994, -	311	936	4	35192.57	5.31	Cytoplasmic
AH14G39150.1	*AhOMT72*	Chr14, 129304986…129307172, -	311	936	3	35207.56	5.83	Cytoplasmic
AH14G43190.1	*AhOMT73*	Chr14, 132761740…132764130, -	359	1080	3	40177.73	6.37	Cytoplasmic
AH14G43200.1	*AhOMT74*	Chr14, 132775330…132777397, +	327	984	2	36928.61	5.51	Cytoplasmic
AH14G43220.1	*AhOMT75*	Chr14, 132793894…132796027, +	362	1089	2	41090.29	4.86	Cytoplasmic
AH14G43240.1	*AhOMT76*	Chr14, 132813746…132815906, +	379	1140	2	42884.6	5.28	Cytoplasmic
AH14G43250.1	*AhOMT77*	Chr14, 132824810…132826734, +	289	870	2	32424.1	4.85	Cytoplasmic
AH14G43260.1	*AhOMT78*	Chr14, 132831217…132831999, +	260	783	1	29079.49	6.03	Extracellular/Cytoplasmic/PlasmaMembrane
AH14G44010.1	*AhOMT79*	Chr14, 133523795…133526394, +	369	1110	3	41613.79	5.55	Cytoplasmic
AH14G44020.1	*AhOMT80*	Chr14, 133543553…133545010, +	263	792	2	29659.24	5.27	Cytoplasmic
AH14G44040.1	*AhOMT81*	Chr14, 133578635…133581542, +	363	1092	3	41374.11	5.83	Cytoplasmic/PlasmaMembrane
AH14G44050.1	*AhOMT82*	Chr14, 133600044…133603205, +	367	1104	3	41974.4	5.39	Cytoplasmic
AH14G44230.1	*AhOMT83*	Chr14, 133827191…133829559, -	369	1110	3	41590	5.52	Cytoplasmic
AH15G03640.1	*AhOMT84*	Chr15, 5904808…5904981, +	57	174	1	6537.49	4.5	Cytoplasmic/Nuclear
AH15G09730.1	*AhOMT85*	Chr15, 17072781…17073111, -	80	243	1	8857.37	6.38	Cytoplasmic
AH15G09740.1	*AhOMT86*	Chr15, 17085732…17086870, -	232	699	2	25463.92	5.59	Cytoplasmic
AH15G30330.1	*AhOMT87*	Chr15, 143877154…143880313, -	231	696	5	25743.82	5.1	Cytoplasmic
AH15G34850.1	*AhOMT88*	Chr15, 149516849…149520694, -	372	1119	2	41633.72	5.41	Cytoplasmic
AH16G14480.1	*AhOMT89*	Chr16, 24990946…24992363, -	283	852	3	30936.49	5.33	Cytoplasmic
AH17G11080.1	*AhOMT90*	Chr17, 17572788…17574790, +	230	693	4	25641.62	6.7	Cytoplasmic
AH17G11130.1	*AhOMT91*	Chr17, 17599988…17615368, -	367	1104	4	40666.91	5.31	Cytoplasmic
AH17G11160.1	*AhOMT92*	Chr17, 17675441…17680659, +	373	1122	3	41174.51	5.31	Cytoplasmic
AH17G11170.1	*AhOMT93*	Chr17, 17720906…17725507, +	373	1122	4	41157.32	5.16	Cytoplasmic
AH17G11190.1	*AhOMT94*	Chr17, 17820754…17838864, +	374	1125	4	41252.7	5.62	Cytoplasmic
AH17G11220.1	*AhOMT95*	Chr17, 17982468…17985690, +	367	1104	4	40481.65	5.71	Cytoplasmic
AH17G11350.1	*AhOMT96*	Chr17, 18600583…18601386, -	267	804	1	29461.77	5.43	Cytoplasmic/Chloroplast
AH17G12150.1	*AhOMT97*	Chr17, 21159366…21161312, +	363	1092	2	40940.59	5.75	Cytoplasmic
AH17G12180.1	*AhOMT98*	Chr17, 21347129…21349187, +	349	1050	2	39358.41	5.19	Cytoplasmic
AH17G12210.1	*AhOMT99*	Chr17, 21390326…21393847, +	363	1092	2	40924.45	5.53	Cytoplasmic
AH17G12230.1	*AhOMT100*	Chr17, 21541743…21545356, -	259	780	2	29208.88	5.3	Cytoplasmic
AH17G12310.1	*AhOMT101*	Chr17, 21840489…21842905, -	288	867	2	32635.6	4.87	Cytoplasmic
AH17G12370.1	*AhOMT102*	Chr17, 21929588…21931865, -	376	1131	2	42715.59	5.29	Cytoplasmic
AH17G12380.1	*AhOMT103*	Chr17, 21978612…21984311, -	384	1155	2	42688.46	6.38	PlasmaMembrane/Cytoplasmic
AH17G12420.1	*AhOMT104*	Chr17, 22160786…22163384, -	352	1059	2	39265.49	5.16	Cytoplasmic
AH17G12450.1	*AhOMT105*	Chr17, 22249751…22251901, -	364	1095	2	40704.96	5.3	Cytoplasmic
AH18G08980.1	*AhOMT106*	Chr18, 10575268…10579544, -	290	873	3	32434.37	5.94	Cytoplasmic
AH18G18630.1	*AhOMT107*	Chr18, 42350728…42358246, -	357	1074	4	39647.35	6.08	Cytoplasmic
AH18G19640.1	*AhOMT108*	Chr18, 53503322…53506529, +	311	936	4	34338.74	9.06	Chloroplast/Mitochondrial
AH19G00660.1	*AhOMT109*	Chr19, 514524…516286, -	362	1089	3	40364.32	5.66	Cytoplasmic
AH19G24900.1	*AhOMT110*	Chr19, 113810989…113811162, -	57	174	1	6537.49	4.5	Cytoplasmic/Nuclear
AH20G07220.1	*AhOMT111*	Chr20, 9290110…9291284, -	361	1086	1	40833.25	5.6	Cytoplasmic
AH20G13670.1	*AhOMT112*	Chr20, 21608191…21610458, +	248	747	5	27947.09	5.83	Cytoplasmic
AH20G19630.1	*AhOMT113*	Chr20, 57791932…57794102, -	248	747	3	27931	5.53	Cytoplasmic
AH20G22290.1	*AhOMT114*	Chr20, 99007639…99013440, -	403	1212	4	44371.69	6.89	Cytoplasmic
AH20G24820.1	*AhOMT115*	Chr20, 114186636…114189506, -	360	1083	3	40458.19	5.68	Cytoplasmic
AH20G24830.1	*AhOMT116*	Chr20, 114193140…114198405, -	360	1083	4	39987.05	6.16	Cytoplasmic

The + and - represents the positive and negative DNA strands.

The subcellular localization prediction of *AhOMT* proteins showed a diverse kind of localization. The main organelle where all *OMTs* were localized was the cytoplasm, while some *AhOMTs* were also localized in more than one cell compartment, including the nucleus, mitochondria, chloroplast, plasma membrane, and extracellular spaces. The physicochemical properties of *AhOMTs* are given in detail in **Table 1**. Similar patterns of genomic and physicochemical properties were found in the *AdOMTs* and *AiOMTs*. The shortest of *AdOMTs* was *AdOMT25* and *AdOMT41*, with a protein and CDS length of 104 aa and 312 bp, respectively. While the longest *AdOMT* was *AdOMT57*, with a protein and CDS length of 1760 aa and 5280 bp, respectively. The other physiochemical properties also varied, as the molecular weight ranged from 11.78 kDa for *AdOMT41* to 194.78 kDa for *AdOMT57*. The theoretical isoelectric points varied from 4.86 for *AdOMT43* to 8.51 for *AdOMT46*. The protein, CDS lengths, and physiochemical properties of *AdOMTs* are given in **Supplementary Table 2**. *OMTs* of *A. ipaensis* also possessed similar protein, CDS lengths and other properties. Proteins varied from 68 aa (*AiOMT43*) to 707 aa (*AiOMT63*), while CDS lengths from 204 bp (*AiOMT43*) to 2121 bp (*AiOMT63*). The expected molecular weight for *AiOMTs* ranged from 7.83 kDa (*AiOMT43*) to 78.87 kDa (*AiOMT63*), while the pI varied from 4.56 (*AiOMT19*) to 9.08 (*AiOMT57*). Most *AiOMTs* were located in the cytoplasm, while others were located in mitochondria, endoplasmic reticulum, and nucleus. **Supplementary Table 3** shows detailed information about *AiOMTs*.

### Phylogenetic relations of *AhOMT* genes

**Figure 1:**
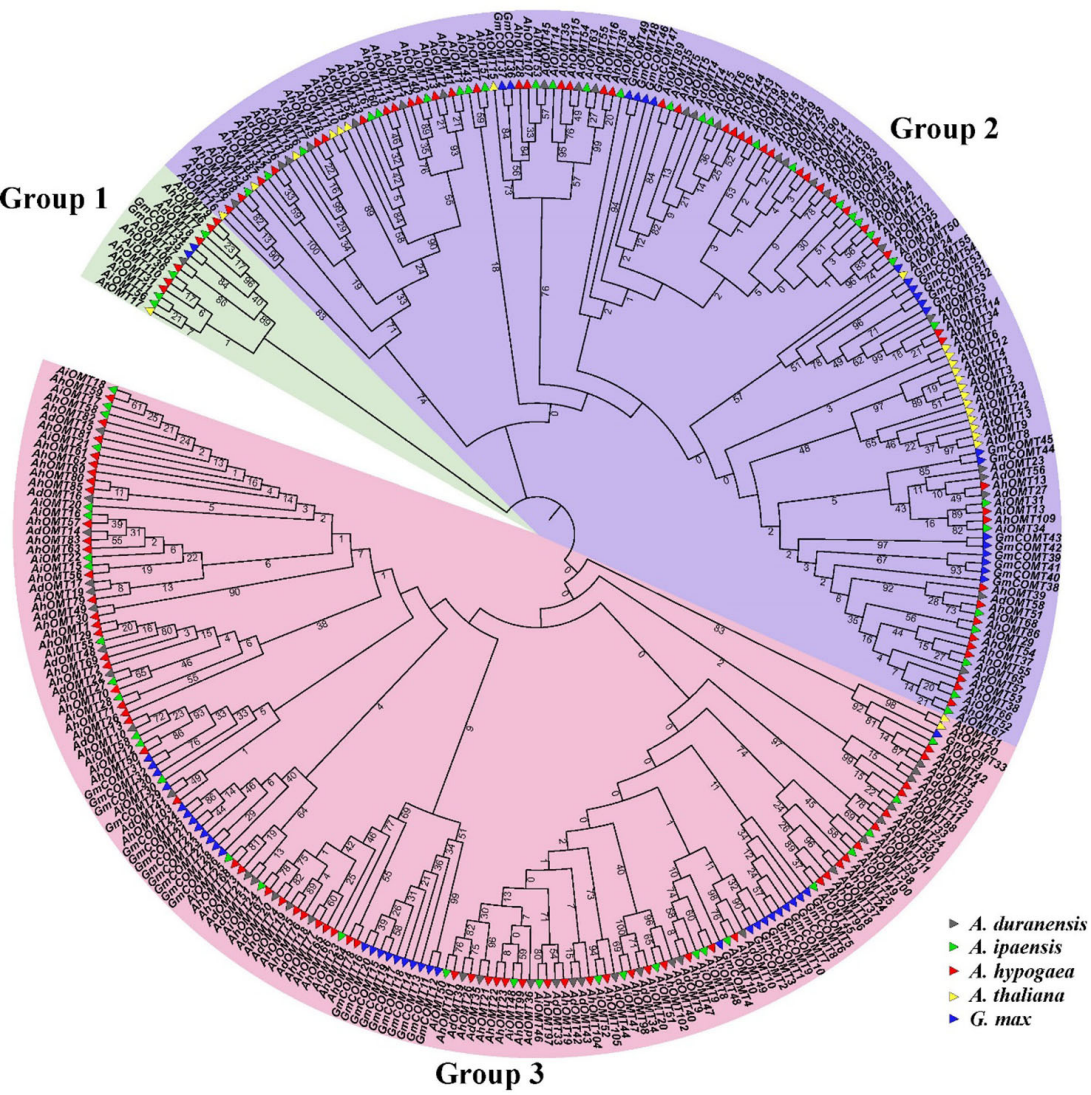
Phylogenetic relationships of O-Methyltransferase genes of *A. hypogea*, *A. duranensis*, *A. ipaensis*, *A. thaliana*, and *G. max*. The phylogenetic tree classified all *OMTs* into three groups, *OMTs* of all species are present in all clades. Group one is the smallest group as compared to other groups.

The phylogenetic tree containing *A. ipaensis*, *A. duranensis*, *G. max*, *A. thaliana*, and *A. hypogaea OMT*s divided them into three main groups (**Figure 1**). *OMTs* of all five species were dispersed in all clades of the phylogenetic tree, indicating that the *OMTs* genes diverged before the divergence of ancestral species. The phylogenetic results revealed that Group I comprised 14 *OMT* members (two *GmOMTs*, one *AtOMT*, four *AiOMTs*, six *AhOMTs*, and one *AdOMT*). Group II comprises 146 *OMT* members (20 *GmOMTs*, 21 *AtOMT*, 31 *AiOMTs*, 50 *AhOMTs*, and 24 *AdOMTs*). Group III contains 160 *OMTs* members (32 from *G. max*, two from *A. thaliana*, 31 from *A. ipaensis*, 62 from *A. hypogaea*, and 33 from *A. duranensis*). In summary, it can be hypothesized from the phylogenetic groupings that *OMTs* from different species with falling in a similar clade will probably perform similar functions. The greater number of *OMTs* in cultivated peanut than in its diploid progenitors and other model plants represent a high evolutionary rate in *A. hypogaea*.

### Chromosomal locations and gene duplication

**Figure 2:**
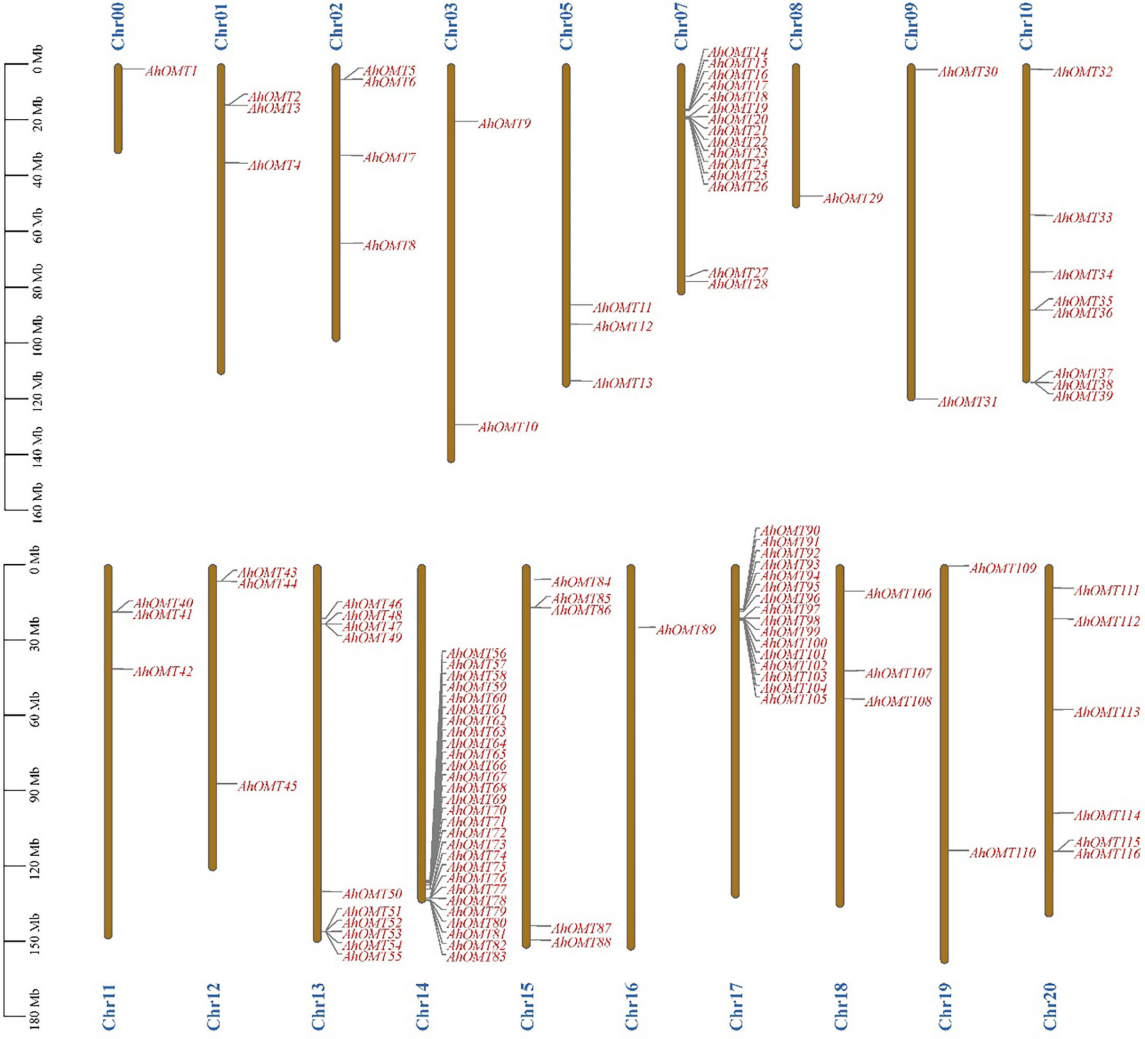
Chromosomal distribution of *AhOMTs* genes. The highest number of *AhOMTs* on A-subgenome were present on Chr07 (15 genes) and in B-subgenome on Chr14 (28 genes) and Chr17 (16 genes), and all other chromosomes possessed varying numbers of OMT genes.

Chromosomal location results revealed that all 116 *AhOMT* genes were dispersed on 18 chromosomes. Chromosomes Chr04 and Chr06 did not possess any *OMT* gene, while one gene was present on the unassembled genome region (Chr00). Chromosomes Chr00, Chr08, and Chr16 possessed one *OMT* each, while Chr07 possessed the highest genes in the A subgenome (15 genes) and in the B subgenome on Chr14 (28 genes) and Chr17 (16 genes), and all other chromosomes possessed varying numbers of *OMT* genes (**Figure 2**). Chromosomes Chr03, Chr09, and Chr19 had two genes each. Chr01, Chr05, Chr11, Chr12, and Chr18 possessed three genes each, Chr02 possessed four, and Chr15 possessed five *AhOMTs*. Chr20 is next with six genes, Chr10 with eight genes, and Chr13 with ten genes (**Figure 2**). The *A. duranensis* genome possessed 58 *OMTs* (*AdOMTs*) unevenly distributed on all ten chromosomes. Only chromosome A09 possessed a single *OMT*; all other chromosomes contained multiple copies of *AdOMTs* ranging from 2-19. Chromosome A08 possessed two *AdOMTs*, while the highest number was present on chromosome A07, which had 19 *AdOMTs* (**Supplementary Figure 1**). The genome of *A. ipaensis* contained 68 copies of *OMT* genes (*AiOMTs*) ranging from 2-16 genes. Chromosome B06 had the least number of *AiOMTs* (two), while chromosomes B04 and B07 possessed the highest number of *AiOMTs* (16 genes each) (**Supplementary Figure 2**).

**Figure 3:**
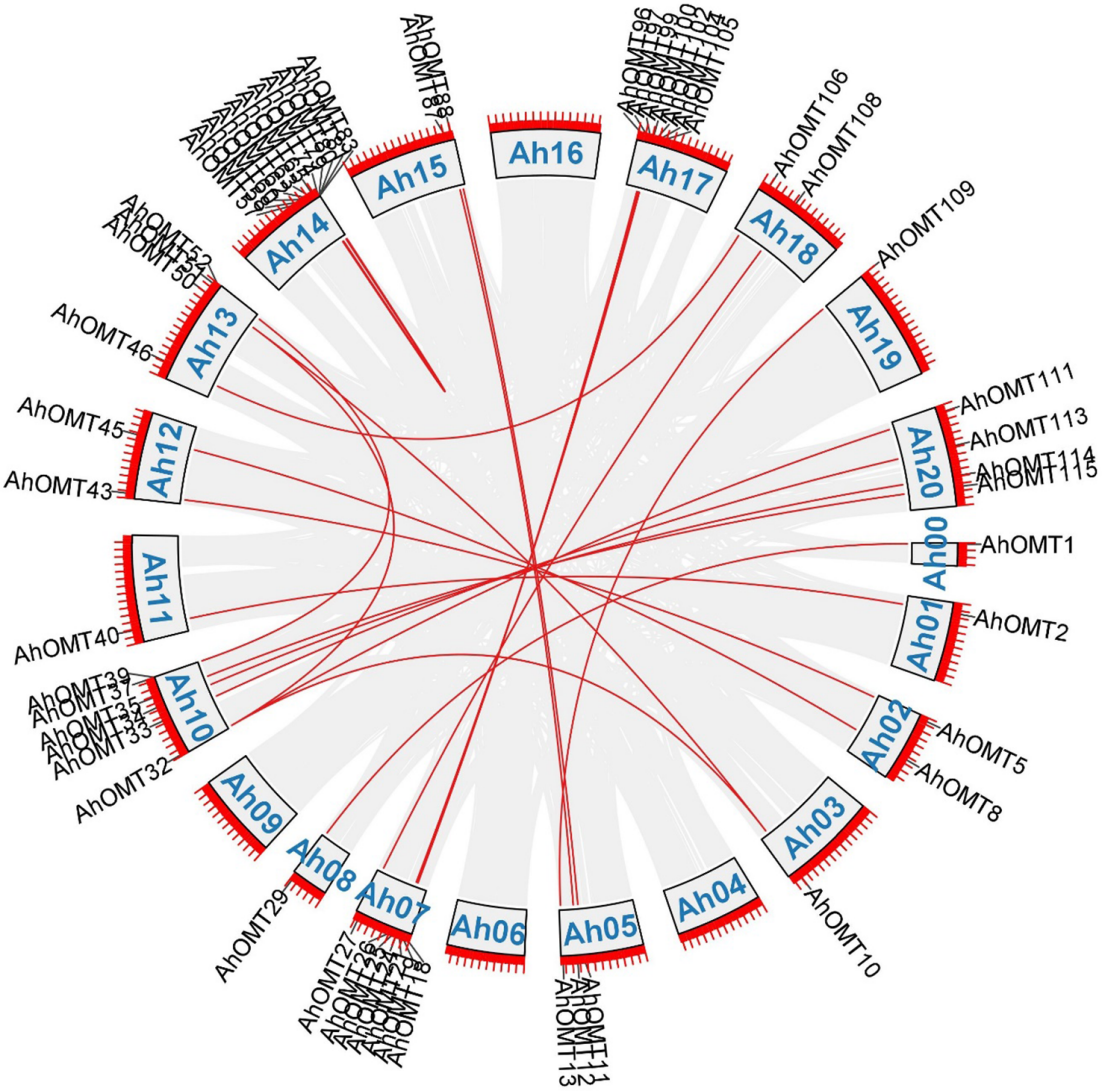
Duplicated gene pairs among *AhOMTs*. Out of 116 genes, 42 are duplicated genes. Red lines show the duplicated *AhOMT* pairs, while the gray lines in the background show the syntenic blocks (duplicated pairs) among different chromosomes.

Gene duplication analysis revealed 32 duplicated pairs of *AhOMTs*. To estimate the molecular evolution rate, the synonymous (Ks) and nonsynonymous (Ka) substitutions were computed for duplicated genes. Positive selection pressure was assumed when Ka/Ks>1, purifying selection when Ka/Ks<1, and neutral selection when Ka/Ks=1 (Yang and Bielawski, 2000). Results showed that mainly purifying selection drove the genome duplication. Furthermore, the duplicated gene pair divergence timeframe was estimated as t=ks/2r. The expected divergence time varied from 1.078 million years ago (mya) for *AhOMT10*:*AhOMT50* to 185.317 MYA for *AhOMT10*:*AhOMT32* (**Table 2**). Most genes were segmentally duplicated, but some were tandemly duplicated (**Figure 3**).

**Table 2:** Calculation of Ka/Ks values and divergence time of duplicated genes.

Seq_1	Seq_2	Ka	Ks	Ka_Ks	Selection Pressure	Divergence Time
*AhOMT2*	*AhOMT40*	0.006822	0.030554	0.223279	Purifying	1.881
*AhOMT5*	*AhOMT43*	0.022377	0.036767	0.608621	Purifying	2.264
*AhOMT8*	*AhOMT45*	0.003812	0.051412	0.074145	Purifying	3.166
*AhOMT10*	*AhOMT32*	0.418478	3.009543	0.13905	Purifying	185.317
*AhOMT10*	*AhOMT50*	0.006978	0.017506	0.398609	Purifying	1.078
*AhOMT11*	*AhOMT88*	0.006941	0.05584	0.124305	Purifying	3.438
*AhOMT12*	*AhOMT87*	0.011542	0.030155	0.382771	Purifying	1.857
*AhOMT13*	*AhOMT109*	0.016391	0.026532	0.617772	Purifying	1.634
*AhOMT18*	*AhOMT96*	0.021194	0.046102	0.459725	Purifying	2.839
*AhOMT19*	*AhOMT97*	0.02151	0.107486	0.200115	Purifying	6.619
*AhOMT19*	*AhOMT102*	0.125147	0.476578	0.262594	Purifying	29.346
*AhOMT21*	*AhOMT99*	0.0749	0.191072	0.391998	Purifying	11.766
*AhOMT21*	*AhOMT104*	0.134389	0.50998	0.263517	Purifying	31.403
*AhOMT24*	*AhOMT105*	0.186122	0.728876	0.255355	Purifying	44.882
*AhOMT25*	*AhOMT100*	0.026577	0.097381	0.272919	Purifying	5.996
*AhOMT26*	*AhOMT102*	0.141688	0.460419	0.307738	Purifying	28.351
*AhOMT27*	*AhOMT108*	0.008499	0.037275	0.228001	Purifying	2.295
*AhOMT29*	*AhOMT1*	0.0036	0.045451	0.079214	Purifying	2.799
*AhOMT32*	*AhOMT50*	0.412074	2.671299	0.15426	Purifying	164.489
*AhOMT32*	*AhOMT111*	0.005889	0.030978	0.190114	Purifying	1.908
*AhOMT33*	*AhOMT113*	0.005231	0.024123	0.216852	Purifying	1.485
*AhOMT34*	*AhOMT114*	0.002375	0.024228	0.098039	Purifying	1.492
*AhOMT35*	*AhOMT115*	0.004795	0.046675	0.102727	Purifying	2.874
*AhOMT37*	*AhOMT52*	0.124998	0.287727	0.434431	Purifying	17.717
*AhOMT39*	*AhOMT51*	0.005973	0.029436	0.202899	Purifying	1.813
*AhOMT46*	*AhOMT106*	0.125462	1.269437	0.098833	Purifying	78.167
*AhOMT57*	*AhOMT83*	0.018462	0.054159	0.340895	Purifying	3.335
*AhOMT58*	*AhOMT80*	0.061586	0.172839	0.356321	Purifying	10.643
*AhOMT60*	*AhOMT81*	0.022063	0.060556	0.364345	Purifying	3.729
*AhOMT62*	*AhOMT80*	0.052874	0.129797	0.407362	Purifying	7.992
*AhOMT63*	*AhOMT79*	0.019671	0.086954	0.226226	Purifying	5.354
*AhOMT65*	*AhOMT74*	0.034348	0.040983	0.838105	Purifying	2.524

### Gene structure and motifs analysis

**Figure 4:**
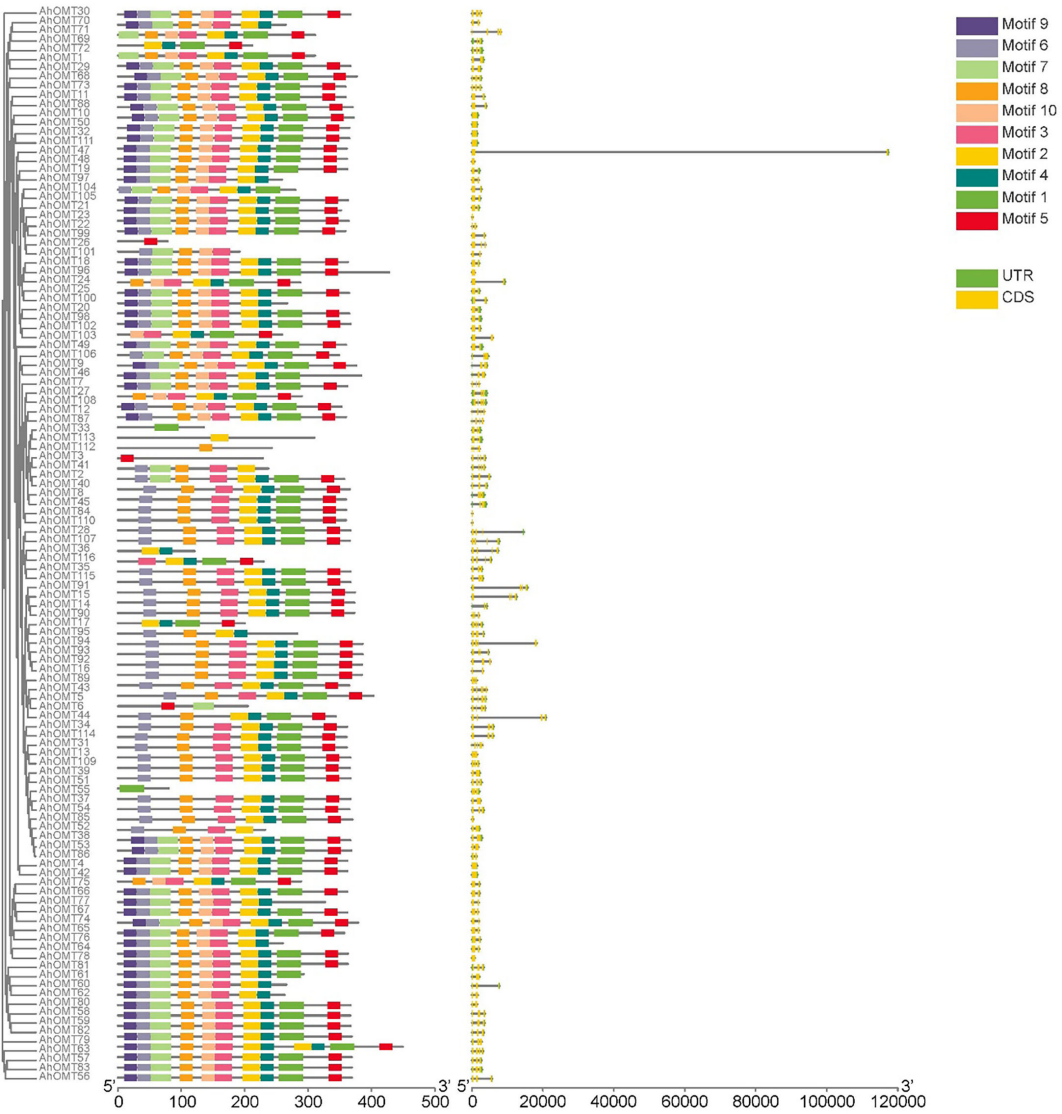
Conserved motifs distribution patterns and gene structure (exons-introns distribution) of *AhOMTs* genes. Commonly shared motifs among genes tend to cluster in the same groups, referring to their similar functions.

To better understand the gene structure of *AhOMTs*, we viewed their exon-intron distribution patterns. According to the findings, the introns in *AhOMT* genes varied from 0 to 5, and exons from 1 to 6. Many *AhOMT* genes were composed of a single intron and two exons. Forty-two out of 116 *AhOMTs* possessed two exons. Three and four exons were also common, as 30 genes possessed three exons while 25 genes had four exons. Thirteen genes were composed of a single exon, and only *AhOMT31* comprised six exons (**Figure 4**). EME server identified conserved motifs inside the full-length protein sequences of *AhOMT* genes in order to determine structural diversification and functional assessment. Ten conserved motifs were predicted in *AhOMT* genes (**Figure 4**). Conserved motifs varied in length as motif 1 was the most extended motif with 39 amino acids, while 4^th^-6^th^ and 8^th^-10^th^ motifs were the shortest with 21 amino acid residues (**Supplementary Table 4**). In a nutshell, conserved motif, phylogenetic, and gene structure analysis indicated that *AhOMT* proteins comprise extremely well-sustained members of amino acids that remain inside a group. Proteins with similar motifs and structures can therefore be functionally related. The motif distribution patterns and gene structure of *OMTs* of wild progenitors were as per *A. hypogaea OMTs*. Information on motifs and structure of *AdOMTs* are given in **Supplementary Figure 3**, and on *AiOMTs* is given in **Supplementary Figure 4**.

### Promoter analysis of *AhOMTs* genes

**Figure 5:**
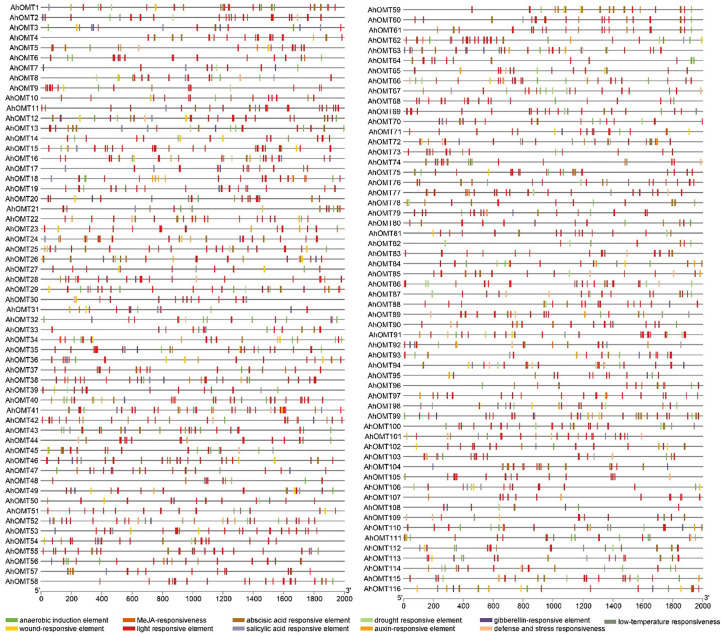
*Cis*-regulatory elements of *AhOMT* promoters. *Cis*-elements analysis revealed important elements responsive to light, hormones, growth and development, and stress responsiveness.

**Figure 6:**
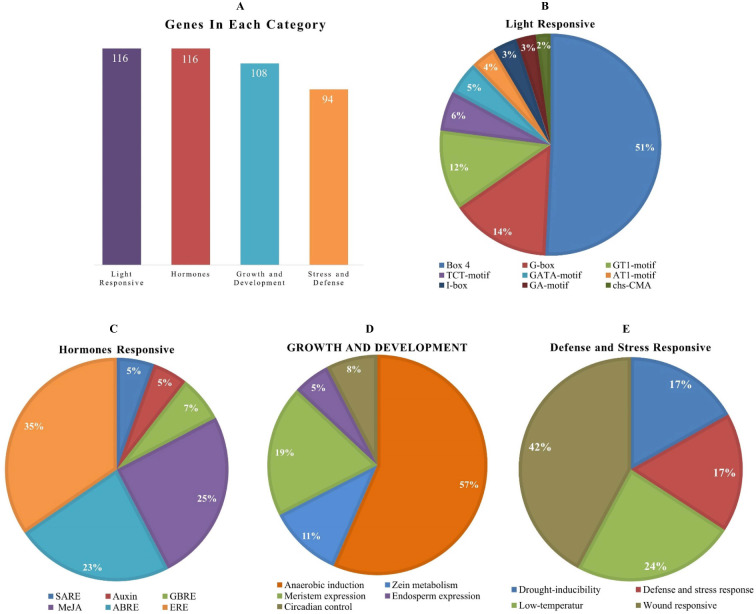
*Cis*-regulatory elements of *AhOMT* promoters. **(A)** The number of genes in different categories of elements. **(B)** Composition of light-responsive, **(C)** hormone-responsive, **(D)** growth and development responsive, and **(E)** stress-responsive factors.

The *cis*-elements of any genes’ promoter are responsible for controlling its expression and functions. We examined *cis*-acting regions in the *AhOMT* promoters to know their functional and regulatory roles. Predicted *cis*-elements showed that aside from the CAAT- and TATA-Box (core promoter elements), a large number of other key elements were also present (**Figure 5**). We classified these *cis*-regulatory elements into four groups according to their functions: development and growth-related, hormones-responsive, light-responsive, and stress-related elements. All 116 *AhOMTs* were enriched with hormones- and light-responsive elements, 108 genes were enriched with growth and development-related elements, and 94 genes were enriched with stress-responsive elements (**Figure 6**).

Elements responsive to light mainly include TCT-motif, GATA-motif, G-box, Box-4, GT1-motif, GA-motif, chs-CMA element, I-box, and AT-1 motif. Other light-responsive elements include 3-AF1 binding site, ATC-motif, AE-box, MRE element, Box II, CAG-motif, CGTCA-motif ATCT-motif, ACE element, Gap-box, TCCC-motif, GTGGC-motif, LAMP-element, LS7 element, and Sp1 element were also present. Hormones responsive class includes ABA-responsive (ABRE), auxin-responsive (AuxRE, AuxRR-core, CGTCA-motif, TGA-box), gibberellins responsive (GARE motif, P- and TATC-box), MeJA-responsive (CGTCA-motif, TGACG-motif), SA-responsive (SARE, TCA-element), and ethylene-responsive (ERE) elements. The growth and development category contained anaerobic induction responsive (ARE), meristem expression responsive (CAT-box), endosperm expression related (GCN4-motif, AACA-motif), circadian control (CAAAGATATC), and zein metabolism-related (O2-site) elements. The stress-responsive class further includes defense and stress response (TC-rich repeats), drought-responsive (MBS), low-temperature responsive (LTR), and wound-related (WUN-motif) elements (**Figure 6**).

### Prediction of miRNAs and synteny analysis

**Figure 7:**
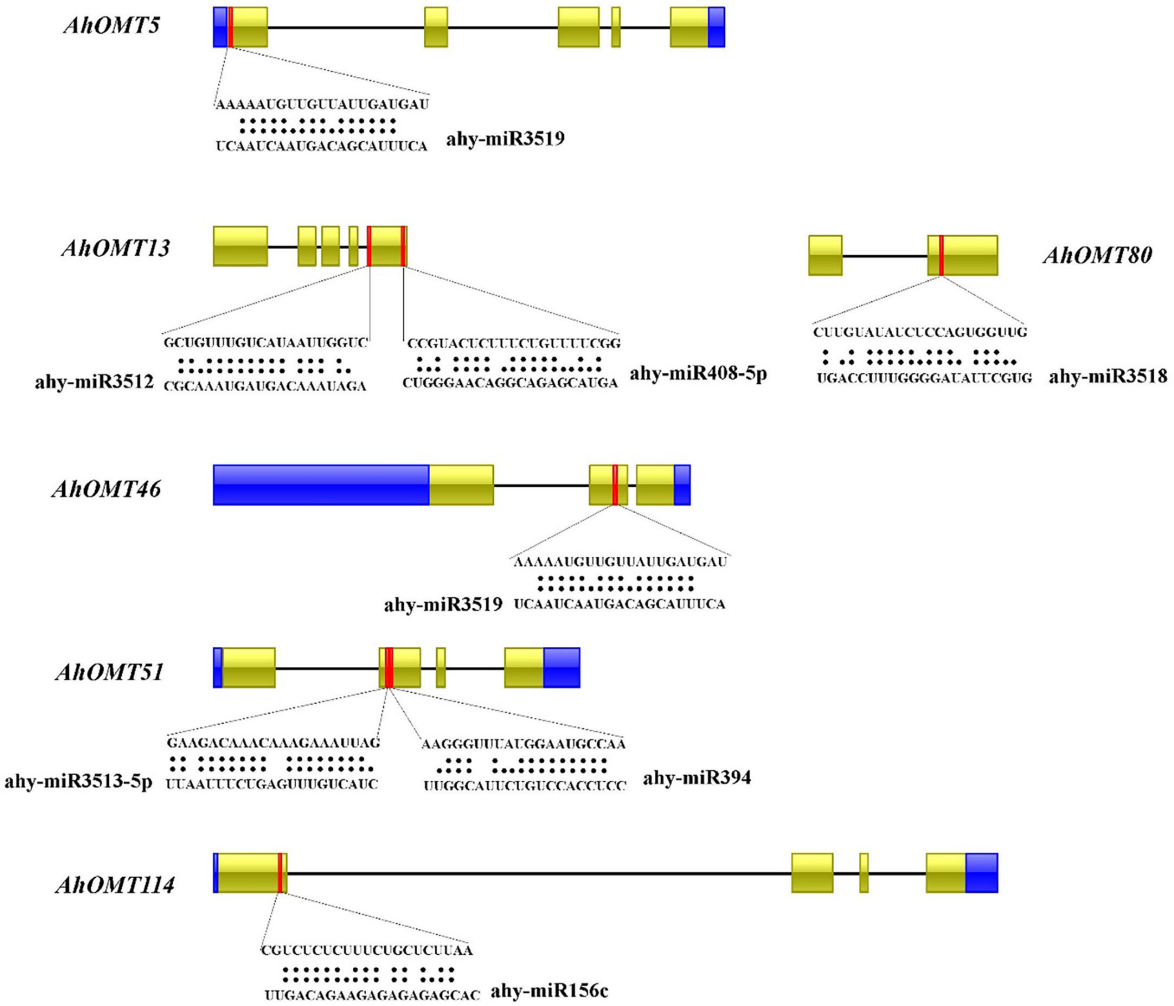
Predicted miRNAs targeting *AhOMTs*. Schematic representation of some miRNAs targeting *AhOMTs*, and their target sites.

Numerous studies in the last few years have revealed that micro-RNAs regulate the expression of genes under developmental processes and stress responses (Chen et al., 2019; Wani et al., 2020; Raza et al., 2021a). For this reason, we predicted *miRNAs* targeting *AhOMT* genes sequentially to get more understanding of miRNA-mediated post-transcriptional regulations of *AhOMT* genes. Micro RNAs from 12 different families targeted 35 *AhOMTs*. **Supplementary Table 5** contains the complete information on all *miRNAs*. Two members of the *miR156* family targeted *AhOMT34*, *AhOMT37*, *AhOMT38*, *AhOMT52-AhOMT55*, *AhOMT87*, and *AhOMT114*. *miR16o-3p* was found to target four *OMTs*. Some of the *miRNAs* targeting the *AhOMTs* with their target sites are shown in **Figure 7**. More research for their expression levels and the genes they target is needed to establish their biological involvement in the peanut genome.

**Figure 8:**
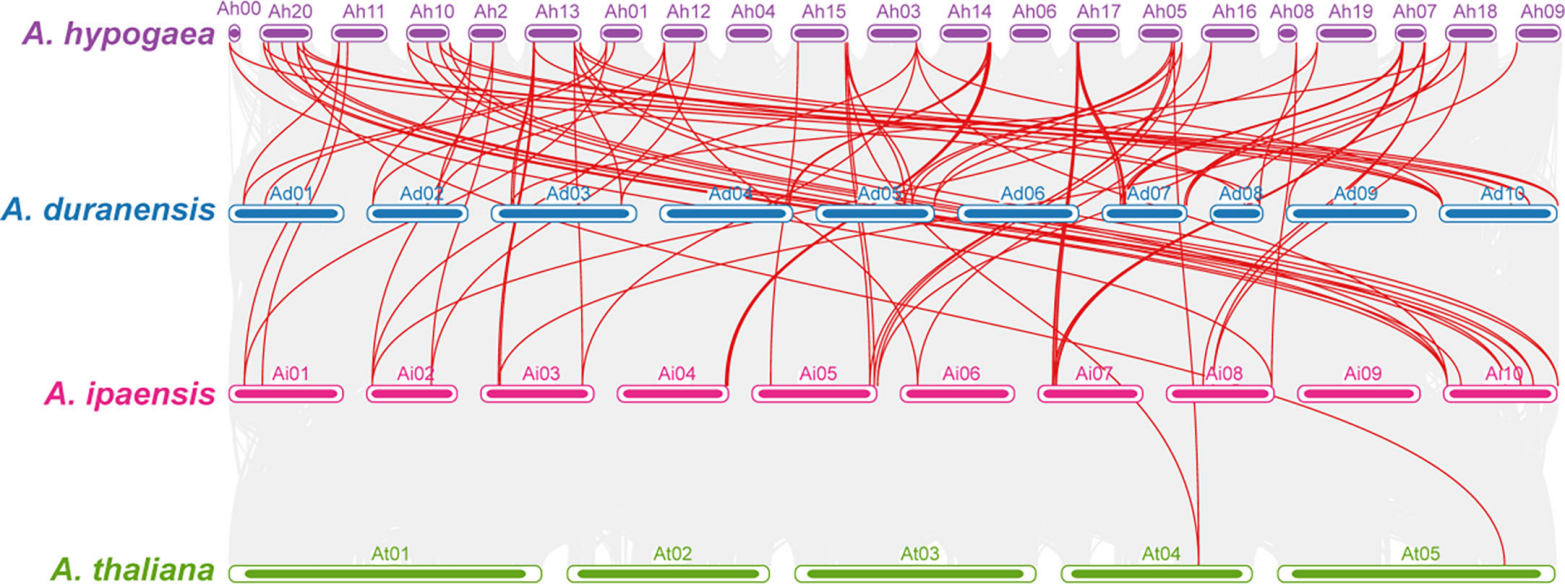
Synteny analysis among *A. hypogea*, *A. duranensis*, *A. ipaensis*, and *A. thaliana*. Synteny analysis showed key evolutionary relationships of *OMTs* in diploid and tetraploid peanut species. *AhOMTs* possessed highly conserved syntenic relationships with other peanut species as compared to *Arabidopsis*.

Comparative synteny analysis among *A. hypogaea*, diploid peanut species, and *A. thaliana* represented remarkable evolutionary, duplication, expression, and functional relationships. *AhOMTs* mainly showed significant syntenic relationships with its wild progenitors and *Arabidopsis*; however, the syntenic relationships of *A. hypogaea* were closer to wild peanut species than *Arabidopsis*. A total of 56 syntenic relationships of *A. hypogaea* were found in the genome of *A. duranensis* and 60 in *A. ipaensis*. In contrast, only four syntenic relationships were found among *AhOMTs* and *AtOMTs*. The synteny analysis showed that *A. hypogaea* is closer to its wild progenitors than *Arabidopsis*. The syntenic relations of these species are shown in **Figure 8**.

### Identification of orthologous gene clusters

**Figure 9:**
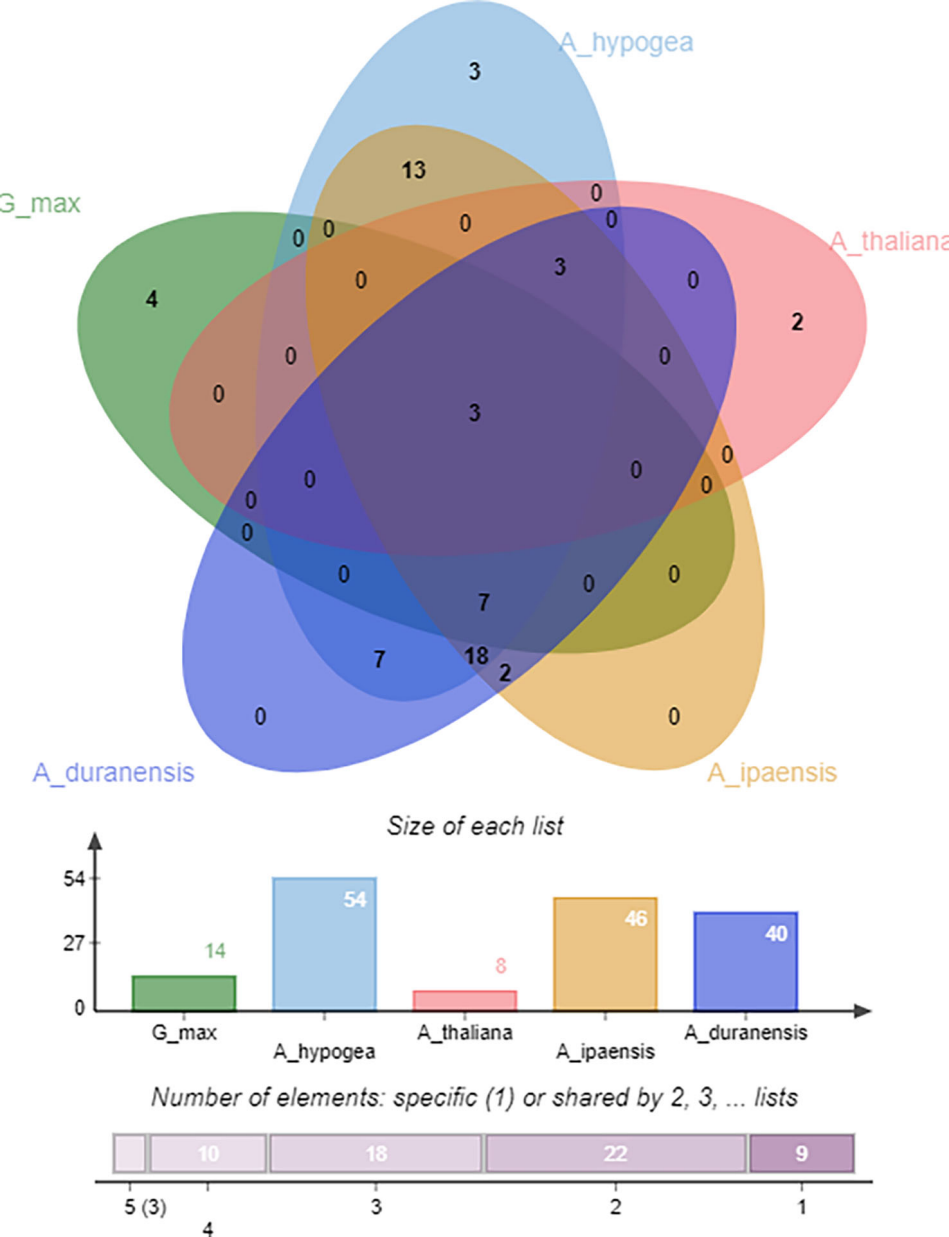
Orthologous genes’ clusters among *A. hypogea*, *A. duranensis*, *A. ipaensis*, *A. thaliana*, and *G. max*. *A. hypogea* recorded maximum clustered. Eighteen gene clusters are solely composed of OMTs found in peanut diploid and tetraploid species, indicating that polyploidization has evolved new peanut-specific orthologous OMT gene clusters.

Identifying orthologous gene clusters is important to assess the polyploidization events during a gene family’s evolution. A relative assessment was developed to identify orthologous gene clusters shared by *A. hypogea, A. duranensis, A. ipaensis, G. max, and A. thaliana*. The detected gene clusters and their respective overlapping regions are presented in greater detail in **Figure 9**. A*. hypogea* recorded maximum clusters, followed by *A. ipaensis*, *A. duranensis*, *G. max*, and *A. thaliana*. Results showed that three gene clusters are shared among all these species, while 18 gene clusters are solely composed of *OMTs* found in peanut diploid and tetraploid species, which indicates that polyploidization has evolved new peanut-specific orthologous *OMT* clusters. We also identified orthologous gene clusters among three peanut species (**Supplementary Figure 5**). Comparatively, 100, 89, 94, 36, and 21 orthologous *OMTs* were found in *A. hypogea*, *A. duranensis*, *A. ipaensis*, *G. max*, and *A. thaliana*, respectively. Thirty in-paralogs were identified in *A. hypogea*, and only two were found in *A. ipaensis*. *A duranensis* did not show any in-paralogous gene. Surprisingly 32, 14, and 20 singletons were also found in *A. hypogea*, *A. duranensis*, and *A. ipaensis*, respectively (**Supplementary Table 6**). Results demonstrated that identified orthologous genes decrease with increased phylogenetic distances.

### Prediction of protein-protein interaction network

**Figure 10:**
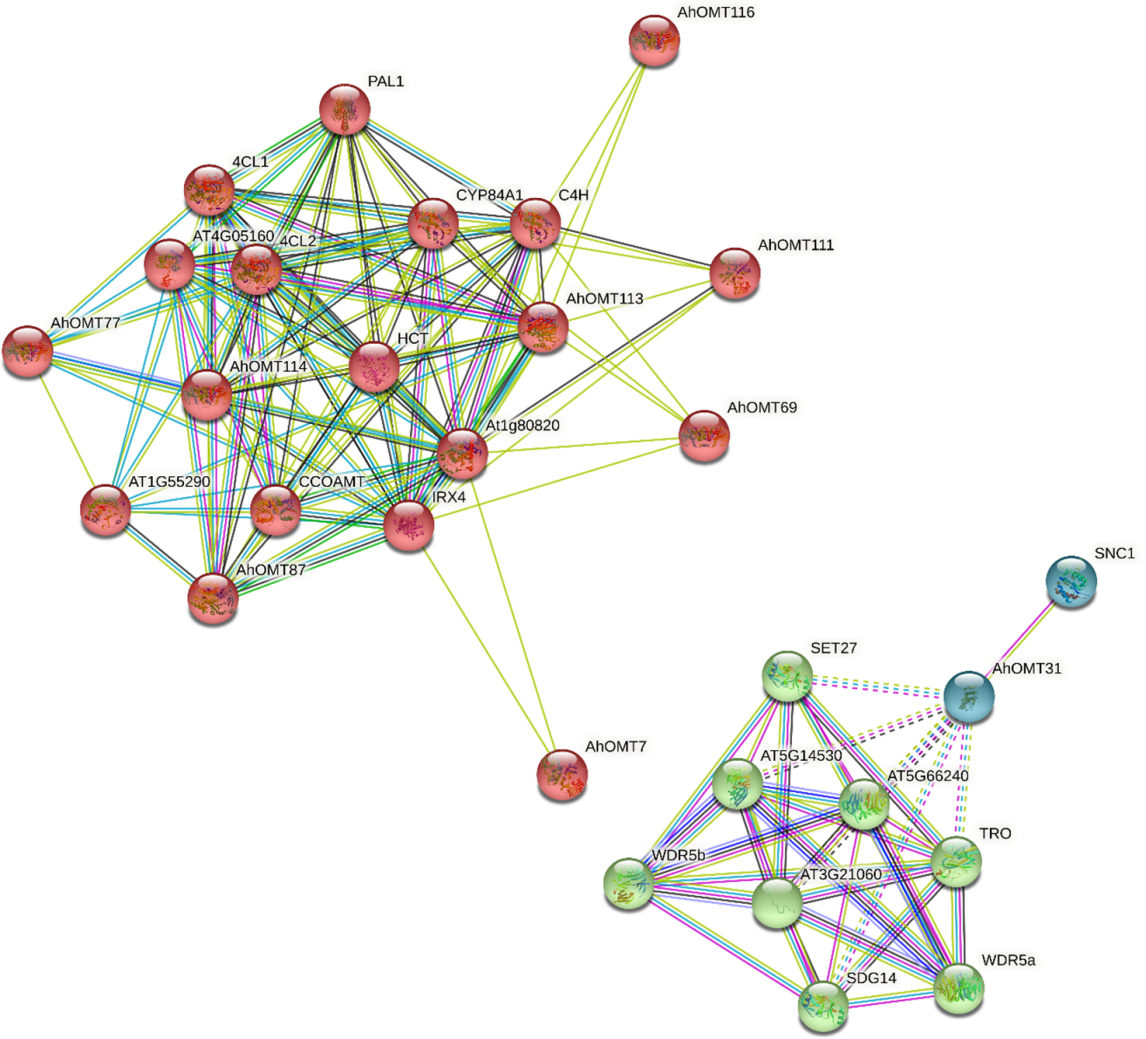
The predicted protein–protein interaction network of AhOMTs using STRING database. Putative protein functions of AhOMTs are predicted on well-studied OMT orthologues in *Arabidopsis*.

The Functions of *AhOMTs* could be speculated based on well-studied *Arabidopsis OMTs*. Using the STRING database, we performed the interaction network analysis of cultivated peanut *OMT* proteins relative to orthologues in *Arabidopsis* to understand their functions. Protein interaction network prediction showed that *AhOMT116* has functions related to C4H that regulate carbon flux to essential pigments for pollination or UV protection. *AhOMT7* and *AhOMT111* may function as Cinnamoyl-CoA reductase 1 (IRX4) involved in lignin biosynthesis at the latter stages. *AhOMT87* has *CCOAMT*-like functions, a putative caffeoyl-CoA O-methyltransferase of *Arabidopsis* that helps in the biosynthesis of feruloylated polysaccharides. *AhOMT77* has 4CL1-related functions (4-coumarate-CoA ligase 1), involved in the later phase of the general phenylpropanoid pathway. *AhOMT31* may function as SNC1, a putative disease-resistance protein of the TIR-NB-LRR-type. The interaction network of *AhOMTs* with well-studied *Arabidopsis* proteins is given in **Figure 10**. Some *OMTs* did not show interactions with reported *Arabidopsis* proteins, and there is a possibility that these proteins have some other functions yet to be reported.

### Functional annotation analysis of *AhOMTs*

**Figure 11:**
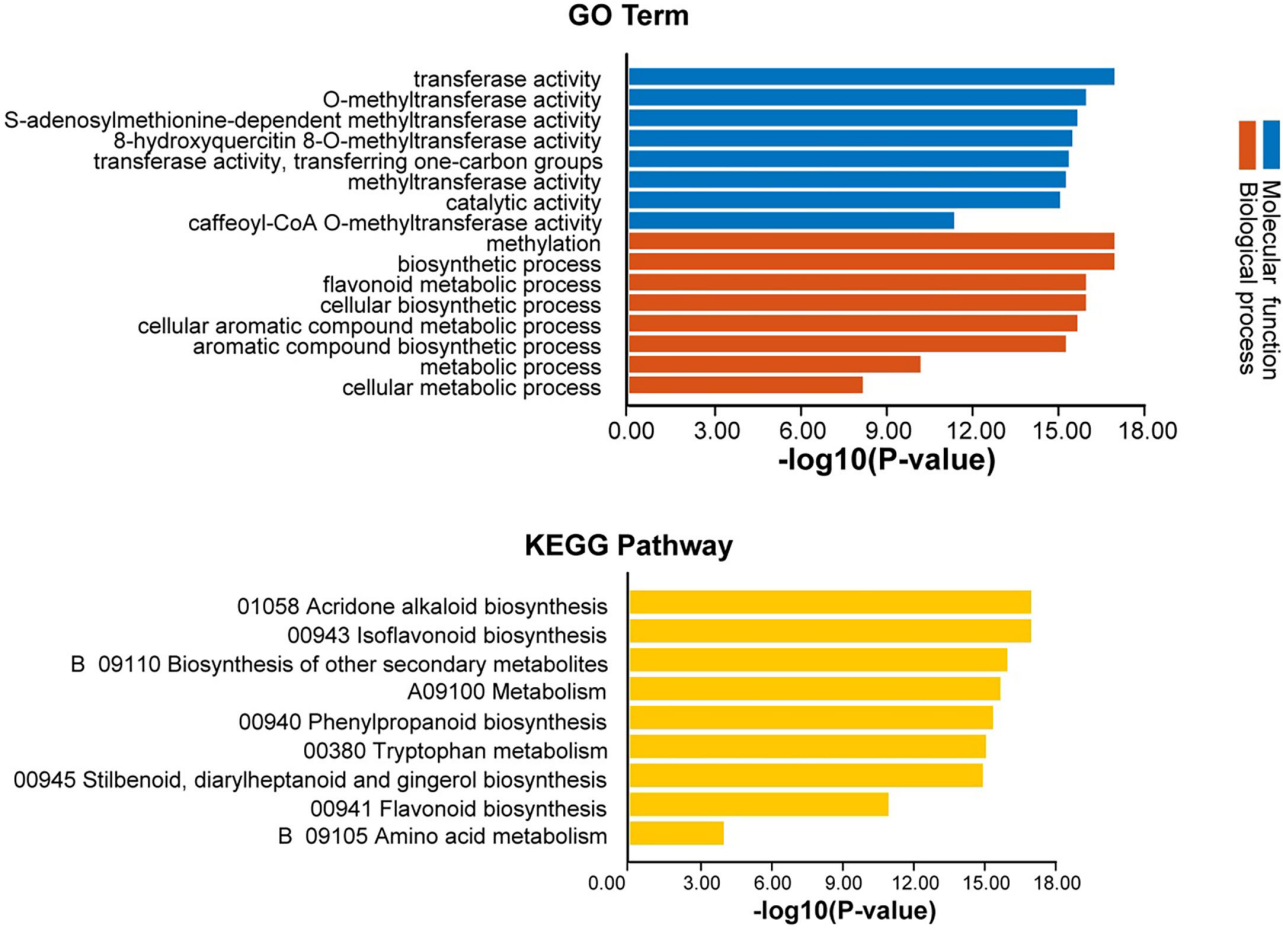
Functional annotation (GO, KEGG) analysis of *AhOMTs*. The gene ontology analysis showed that *AhOMTs* are involved in molecular function (MF), and biological processes (BP). The KEGG enrichment analysis showed that *AhOMTs* are mainly involved in metabolic processes.

GO annotation analysis of *AhOMTs* was performed to view their possible roles in biological processes (BP), molecular functions (MF), and cellular components (CC). GO enrichment results provided highly enriched terms related to BP, MF, and CC (**Figure 11**). *AhOMTs* were mainly involved in MF and BP categories. *AhOMTs* were highly enriched in transferase activity (GO:0016740), catalytic activities (GO:0003824), methyltransferase activity (GO:0008168, GO:0008171, GO:0042409), and S-adenosylmethionine-dependent methyltransferase activity (GO:0008757) in MF category. In the BP category, *AhOMTs* were highly enriched in methylation (GO:0032259), biosynthetic process (GO:0009058, GO:0044249), cellular metabolic processes (GO:0044237, GO:0008152), and aromatic compound metabolism (GO:0006725). The KEGG enrichment analysis showed that *AhOMTs* are mainly involved in metabolic processes, including 01058 acridone alkaloid biosynthesis, 00943 isoflavonoid biosynthesis, B 09110 secondary metabolites biosynthesis, 00380 tryptophan metabolism, 00941 flavonoid production, and amino acid B 09105 metabolism (**Figure 11**). Collectively, it is evident from functional annotation analysis that *AhOMTs* play key roles in several cellular, biological, and molecular functions.

### Expression profiling of *AhOMTs* in different organs

**Figure 12:**
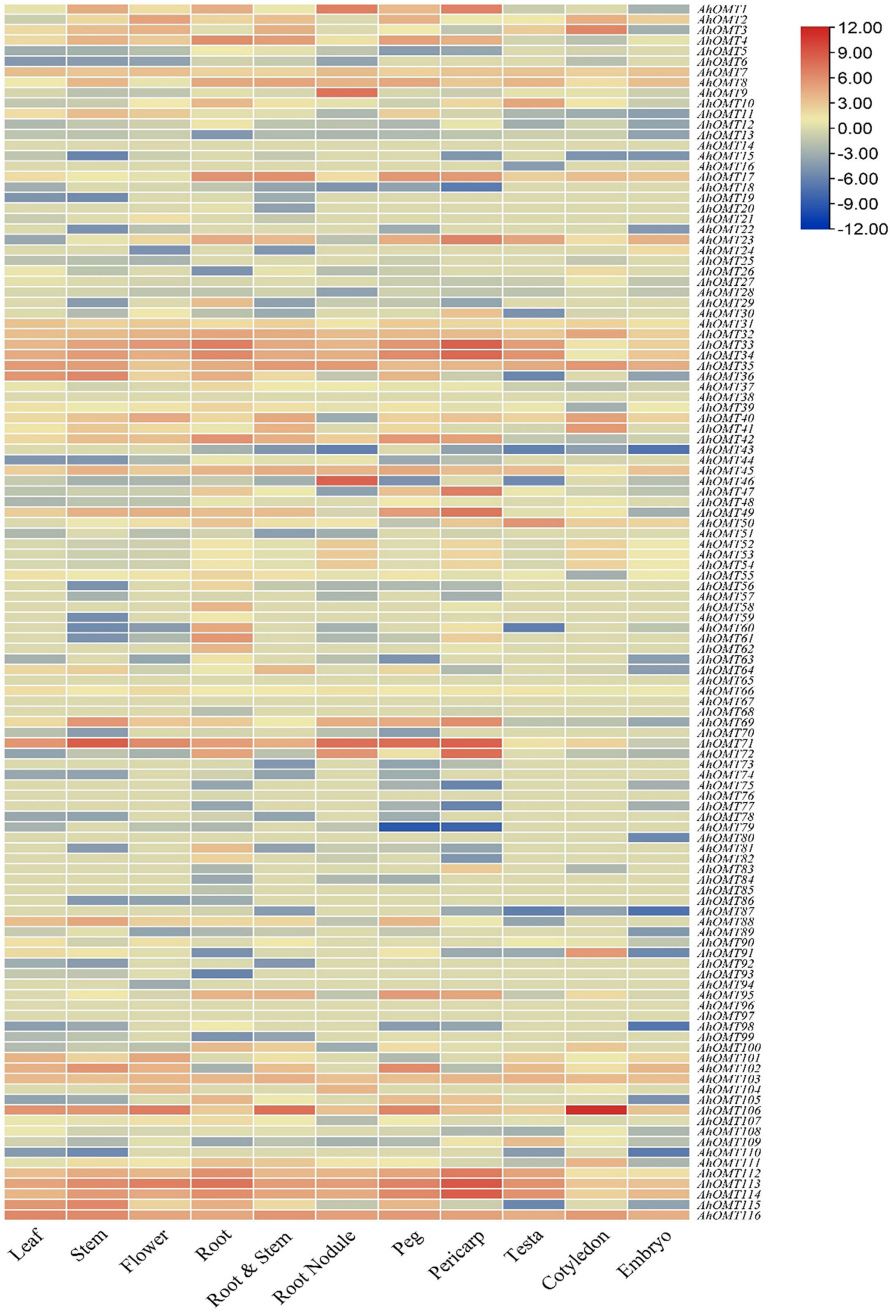
Transcriptome expression of *AhOMTs* in different tissues. *AhOMTs* possessed varying expression matrix in different tissues. All genes possessed varying levels of expression in different tissues.

*AhOMT* genes’ expression levels in different organs/tissues, containing leaf, stem, flower, root, root nodule, peg, pericarp, testa, cotyledon, embryo, etc., was determined using the peanut RNA-seq datasets. According to the expression profiling results, there was a noticeable variance in the expression of various tissues. Transcriptome expression results showed that *AhOMT32-AhOMT35, AhOMT45, AhOMT71, AhOMT106, AhOMT113, AhOMT114*, and *AhOMT116* genes showed relatively higher levels of transcriptional abundance in the leaf, stem, flower, root, root nodule, peg, pericarp, testa, cotyledon, and embryo. These genes can be suitable candidates for improving peanut growth and yield. *AhOMT9* and *AhOMT46* specifically showed high expression in root nodules (**Figure 12**). It can be speculated that these two genes are good targets to improve nitrogen fixation that can provide good crops by effectively fixing the soil nitrogen. FPKM values of transcriptome expression of *AhOMTs* are given in **Supplementary Table 6**.

### Expression profiling of *AhOMTs* under hormones, drought, and temperature stress

**Figure 13:**
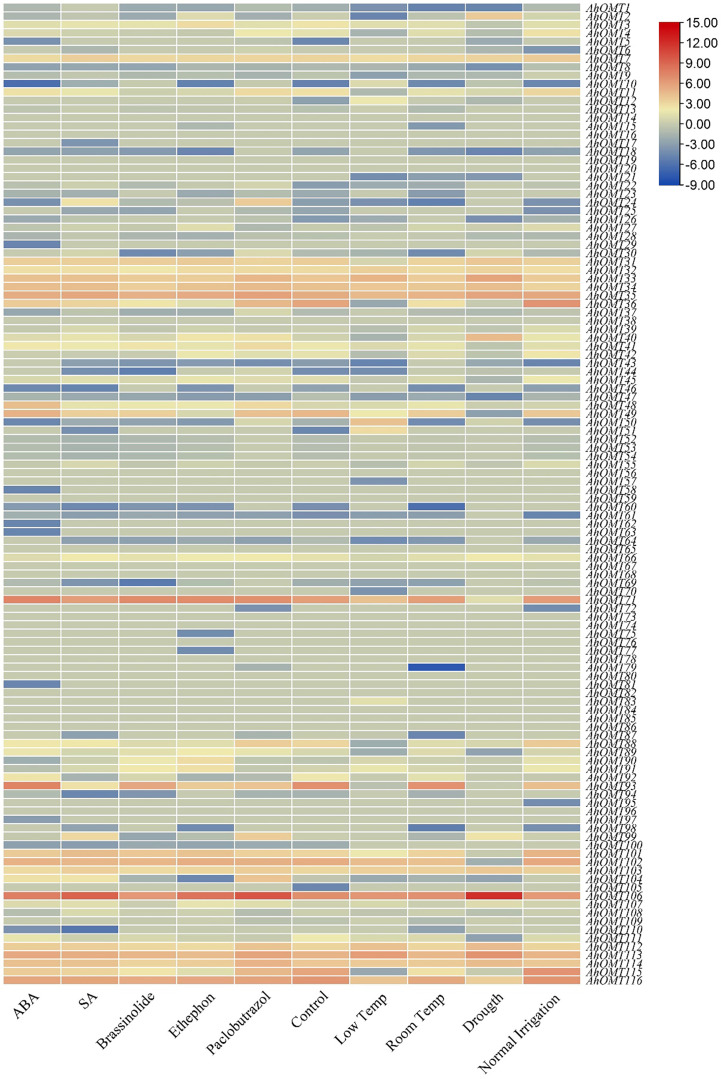
Transcriptome expression of *AhOMTs* under different hormones and stress conditions. Under the stress conditions, the *AhOMT106* gene was most active, while *AhOMT35*, *AhOMT71*, *AhOMT113* were also expressed in most of cases.

Transcriptome data provided the expression patterns of 116 *AhOMTs* for different phytohormones (ABA, SA, Brassinolide, Paclobutrazol, and Ethephon) treatment, water stress (drought and regular irrigation), temperature stress (4°C and 28°C). Under temperature stress, the *AhOMT106* gene was highly active, while *AhOMT35*, *AhOMT71*, and *AhOMT113* were also expressed in most cases, but *AhOMT35* did not show expression under drought stress. Almost 16 genes showed expression in response to ABA and SA, 14 genes responded to brassinolide, and 12 genes were responsive to ethephone. Thirteen genes were expressed under decreased temperature, and almost 11 genes were responsive to drought stress (**Figure 13**). Many genes were non-responsive to the hormones, water and temperature treatments.

### Quantitative expression profiling under ABA and low-temperature treatment

**Figure 14:**
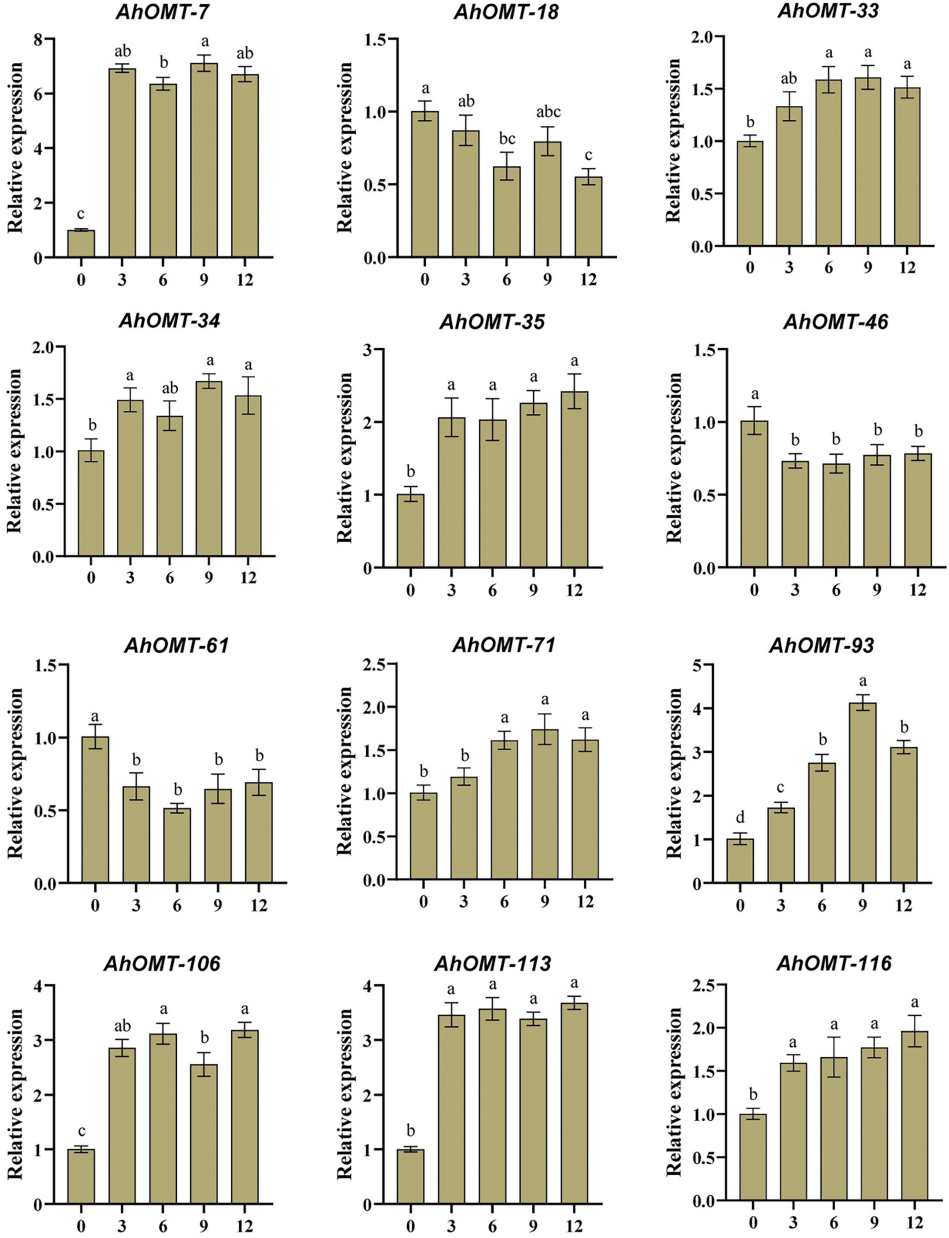
Expression profiling of *AhOMT* genes in response to ABA treatment. Mainly *AhOMT* genes recorded increased expression under ABA stress, while some genes were down-regulated. a, b, and c represents the significance levels among expression at different time points.

**Figure 15:**
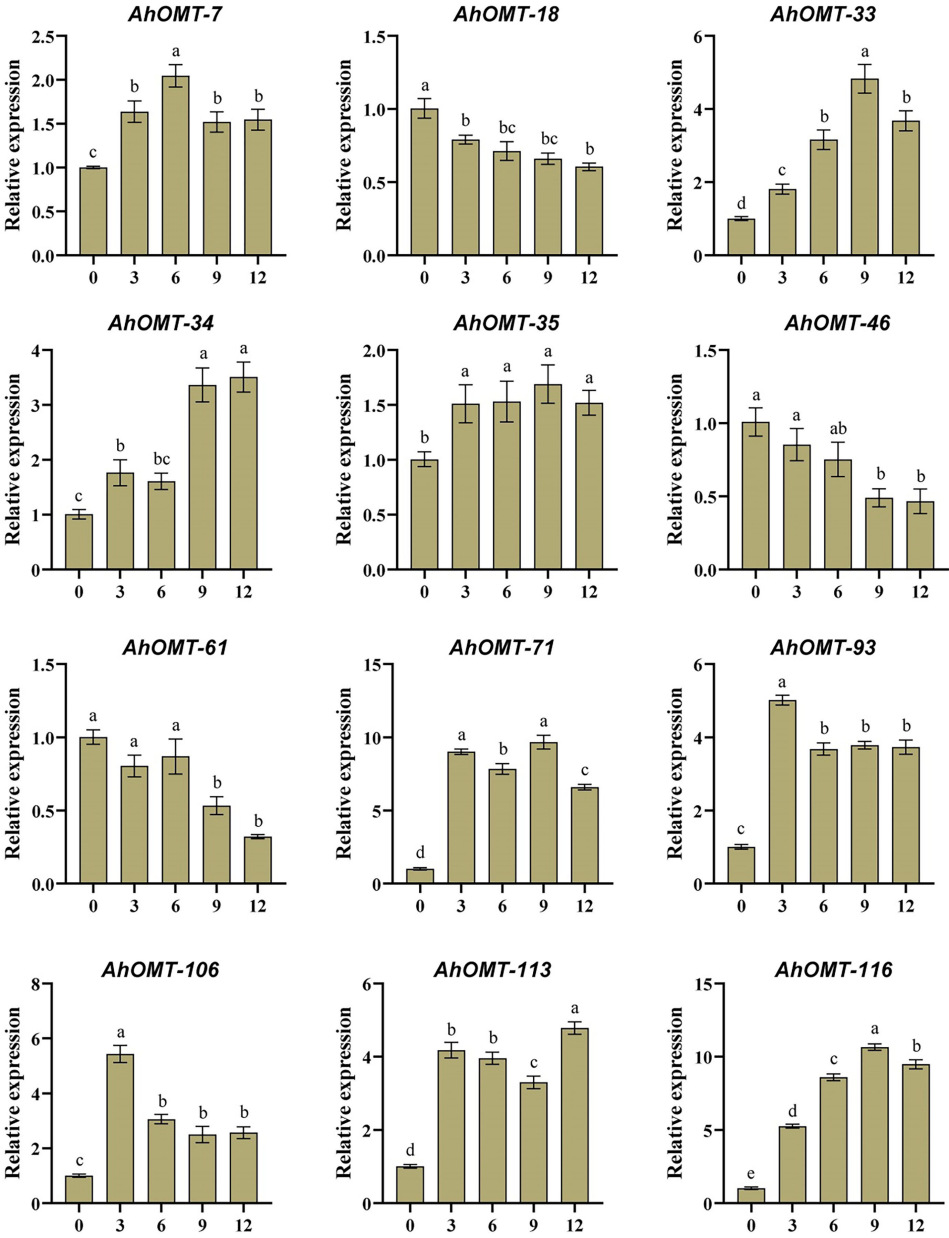
Expression profiling of *AhOMT* genes in response to cold stress. Mainly *AhOMT* genes recorded increased expression under cold stress, while some genes were down-regulated. a, b, and c represents the significance levels among expression at different time points.

For real-time expression profiling by qRT-PCR, 12 *AhOMT* genes were randomly selected. These genes included *AhOMT-7, AhOMT-18, AhOMT-33, AhOMT-34, AhOMT-35, AhOMT-46, AhMT-61, AhOMT-71, AhOMT-93, AhOMT-106, AhOMT-113*, and *AhOMT-116*. These genes were selected based on their response to hormones, water and temperature stress, while genes with higher and lower expression were considered. Under ABA treatment, the expression of all selected genes corresponds to their transcriptome expression. For instance, *AhOMT-7, AhOMT-33, AhOMT-34, AhOMT-35, AhOMT-71, AhOMT-93, AhOMT-106, AhOMT-113*, and *AhOMT-116* were upregulated under ABA stress, while *AhOMT-18*, *AhOMT-46*, and *AhOMT-61* were downregulated (**Figure 14**). Under low temperature, a similar expression was found as of ABA treatment. Although there were some deviations in transcriptome expression and qRT-PCR expression, overall, the expression pattern of all selected genes is in accordance with transcriptome expression (**Figure 15**). The results of qRT-PCR represent the reliability of transcriptome datasets.

## Discussion

Several plants, including *A. thaliana*, *B. distachyon*, *B. napus*, *P. trichocarpa, O. sativa*, and others, have been studied at the whole-genome level to determine the presence and possible roles of *OMT* family genes. Because of their importance for synthesizing S-type lignin, the roles of *OMTs* have been well established. Lignin is the cell wall’s most important component to cope with environmental and biological stress (Boerjan et al., 2003). Reduced lignin production poses the plant to a lodging state (Hu et al., 2015). Reduced lignin concentration in legumes reduces stalk strength which ultimately reduces diseases and pathogens resistance (Bellaloui, 2012). Genome size, genome duplication, and gene distribution all have a significant influence on genetic diversity. Genetic duplication has been recognized for years as a source of the expression, originality, and variety found in gene families across species (Wang et al., 2012). Additionally, some *AhOMT* duplications may be crucial to their multiplication as they can bring neofunctionalization and diversity in gene families (Lavin et al., 2005; Chapman et al., 2008).

Some gene families have originated and extended due to tandem or segmental duplications. Gene family’s evolution in this manner is crucial for their diversification (Cannon et al., 2004). The opposite is also true: gene function may have an impact on copy number and genome structure, resulting in widely disparate patterns of segmental or tandem duplication (Cannon et al., 2004). After tandem duplication, genes occur in clusters (Savard et al., 2011). It is important to understand the evolution of gene clusters to provide updated information on evolutionary history. Previously occurrence of tandem duplication was confirmed in pomegranates by gene mapping by Yuan and coworkers. They identified three *OMT* genes (*PgOMT01* to *PgOMT03*). Relatively large scale duplication of the pomegranate genome resulted in forming the *PgOMT* tandem duplications (Yuan et al., 2018). To a certain extent, tandem duplication has evolved the *PgAOMT* family.

Exon numbers and distribution patterns have a key role in the expression of any gene (Kolkman and Stemmer, 2001). In our investigation, most *AhOMTs* had fewer introns, and members of the same evolutionary group tended to have exon–intron patterns comparable. For instance, the presence of two or more introns in *AhOMT* genes demonstrates that the *OMT* gene development may be directly tied to the diversity of gene architectures. A similar set of findings has also been observed for the *OMT* gene family in Chinese jujube (Song et al., 2017). Several studies have found that genes with lesser introns expressed rapidly as introns can influence expression by delayed transcript synthesis in three different means, by (1) splicing, (2) increasing the length of the growing transcript, or (3) increasing the energy requirement of the transcript of lengthy transcripts (Jeffares et al., 2008). Less number of introns in most *AhOMTs* than its progenitors indicates a possible quicker response to induction; however, additional research is required to confirm this hypothesis. *OMT* proteins from five species used in this study were clustered into three phylogenetic groups. Conferring to the phylogenetic tree, three unique groups represents substrate specificity according to their functional traits (Joshi and Chiang, 1998).

To control gene transcription, various proteins must bind to *cis*-regions of the promoter. GT1-motif (Gao et al., 2004), GATA-motif (Argüello-Astorga and Herrera-Estrella, 1998), I-box (Donald and Cashmore, 1990), and G-box (Giuliano et al., 1988), are *cis*-regions needed for light-mediated transcription. According to our findings, S-type lignin may be controlled by *AhOMT* genes, which may interact with light-induced proteins and have circadian patterns in their gene promoters. The circadian rhythm regulates many genes in higher plants, including those involved in photosynthesis and starch mobilization. Hormones highly influence plant growth and development. According to Kim and coworkers, the kenaf *OMT* gene (*Hibiscus cannabinus*) is expressed after six hours of SA, ABA, auxin, ethylene, and GA treatment (Kim et al., 2013). Their findings also support our results, as *AhOMT* genes were generally influenced by hormone treatments. With this, SA-related factors were discovered in the *AhOMT* promoters, implying their key function in the hormonal regulation of *AhOMT*. When they studied the *OMT* gene, they observed that it could be stimulated by H_2_O_2_, cold, and salt, which showed that hormonal and abiotic stimuli might affect the *OMT* genes’ transcription. We also found similar findings for cold stress, as *AhOMTs* were highly influenced (up- and down-regulated). Another study indicated that *Brassica napus OMT* family genes were more highly expressed under drought-stressed circumstances than in regular irrigation (Li et al., 2016). The cold and drought have been shown to significantly increase the expression of an *OMT* gene in *Ligusticum chuanxiong* (Li et al., 2015). Some *OMT* promoter sequences included stress-related motifs such as ARE, LTR, and MBS. *OMT* genes are influenced by salt, and cold stress (Kim et al., 2013) and the presence of stress-responsive elements suggests that *OMT* genes might play a role in neutralizing the abiotic stresses. Some *AhOMT* gene promoters were revealed to have heat-responsive and MBS sights that can collectively induce drought tolerance. In addition to these CREs, stress response involves TC-rich repeats, W1-BOx, ARE, and LTR (Zhang et al., 2015). In light of these studies, it could be speculated that abiotic stress may promote *AhOMT* genes's expression, although more work is required for its confirmation. Micro-RNAs have got wide attention for their developmental and stress-tolerance roles. We identified miRNAs ahy-miR156a, ahy-miR167-3p, ahy-miR3513-5p, ahy-miR3521, ahy-miR156a, ahy-miR160-3p, ahy-miR3508, ahy-miR3513-3p, ahy-miR3518, ahy-miR3519 etc., targeting *AhOMTs* (**Supplementary Table 5**). ahy-miR3521 have been reported to target the *AhOPT3*.2, this gene is also targeted by ahy-miR156a. additionally ahy-miR156a also targets and down-regulates the *AhOPT3.3* and *AhOPT3.4*. ahy-miR167-3p targets *AhYSL3.2*, *AhYSL3.4*, and *AhYSL3.7*. all of these miRNAs downregulates their corresponding genes by mRNA cleavage (Wang et al., 2022). Our miRNAs prediction results also revealed their cleaving activity.

The *OMT* genes in plants have earlier been shown to be vital genes that regulate the expression of a protein necessary for development and growth (Zhang et al., 2021). Gene expression in different organs and tissues was investigated in this research. *AhOMTs* demonstrated diverse expressions in time- and space-defined manners. The expression differences in different tissues indicate the functional differences between *OMT* genes (Zhang et al., 2021). This research also demonstrated that the expression of these genes might be triggered by a certain environment or may be highly unique to a particular organ or developmental stage. Among various abiotic stresses, low temperature and drought stress significantly impair the plant growth and production (Raza et al., 2021b; Raza et al., 2022a; Raza et al., 2022b; Raza et al., 2023) Owing to this, the *OMT* expression under these stressful environments was investigated. According to our findings, the expression of *AhOMT-7, AhOMT-33, AhOMT-34, AhOM-35, AhOMT-71, AhOM-93, AhOMT106, AhOMT-113*, and *AhOMT116* increased when exposed to low temperatures and hormones treatment. Under drought stress, some *AhOMTs* were up-regulated, and others were down-regulated. Our findings are in agreement with previous reports such as *OMTs* were upregulated in response to drought stress in grape barriers (Giordano et al., 2016) and down-regulated in *Brassica napus* (Li et al., 2016). In terms of the mechanism of this event, further research is needed in this area as well. In the near future, the integration of genomics and genome editing technologies could be coupled to improve the production of orphan crops including peanut (Yaqoob et al., 2023). As a result, evolutionary links, structure, and expression of *AhOMT* genes were thoroughly investigated in this work, revealing that these genes played a critical role in peanut stress tolerance and offered a theoretical basis for peanut breeding efforts.

## Conclusion

This study identified 116 *OMT* genes in cultivated peanut. Sequentially to get well perceptive of the *AhOMT* genes, we conducted a wide range of genomic analyses, including evolutionary and genomic characterization, genes structural analysis, *cis*-acting regions, prediction of *miRNAs*, and conserved motifs analysis. A combination of gene structure and phylogenetic analysis revealed three main groups of *AhOMTs*. In addition, these genes’ expression was profiled across different tissues against low temperature, hormones, and drought stress. Furthermore, the *AhOMT* genes expression demonstrated that *AhOMT-7, AhOMT-33, AhOMT-34, AhOM-35, AhOMT-71, AhOM-93, AhOMT106, AhOMT-113*, and *AhOMT116* played a vital role against low temperature, hormones, and drought treatments. This study establishes the framework for future work into the functional study of *AhOMT* in peanut breeding programs.

## Data availability statement

The datasets presented in this study can be found in online repositories. The names of the repository/repositories and accession number(s) can be found below: https://www.ncbi.nlm.nih.gov/bioproject/PRJNA480120.

## Author contributions

WZ and HC conceived the idea and designed the study. TC, YS, YZ, QY, and XC analyzed the data and wrote the manuscript. KC, YC, MG, HD, YP, AR, and CZ helped in literature search, revision, and provided technical guidance. WZ, HC, and YZ supervised the work and edited the final version. TC and YS equally contributed to the manuscript. All authors contributed to the article and approved the submitted version.

## References

[B1] Argüello-Astorga G. Herrera-Estrella L. (1998). Evolution of light-regulated plant promoters. Annual Review of Plant Physiology and Plant Molecular Biology 49, 525–555. doi: 10.1146/annurev.arplant.49.1.525 15012245

[B2] Bailey T. L. Johnson J. Grant C. E. Noble W. S. (2015). The MEME suite. Nucleic Acids Research 43, W39–W49. doi: 10.1093/nar/gkv416 25953851 PMC4489269

[B3] Bellaloui N. (2012). Soybean seed phenol, lignin, and isoflavones partitioning as affected by seed node position and genotype differences. Food Nutr. Sci. 3, 447. doi: 10.4236/fns.2012.34064

[B4] Bertioli D. J. Cannon S. B. Froenicke L. Huang G. Farmer A. D. Cannon E. K.. (2016). The genome sequences of arachis duranensis and arachis ipaensis, the diploid ancestors of cultivated peanut. Nature Genetics 48, 438–446. doi: 10.1038/ng.3517 26901068

[B5] Bi C. Chen F. Jackson L. Gill B. S. Li W. J. (2011). Expression of lignin biosynthetic genes in wheat during development and upon infection by fungal pathogens. Plant Molecular Biology Reporter 29, 149–161. doi: 10.1007/s11105-010-0219-8

[B6] Boerjan W. Ralph J. Baucher M. (2003). Lignin biosynthesis. Annu. Rev. Plant Biol. 54, 519–546. doi: 10.1146/annurev.arplant.54.031902.134938 14503002

[B7] Bout S. Vermerris W. (2003). A candidate-gene approach to clone the sorghum brown midrib gene encoding caffeic acid O-methyltransferase. Molecular Genetics and Genomics 269, 205–214. doi: 10.1007/s00438-003-0824-4 12756532

[B8] Buer C. S. Imin N. Djordjevic M. A. (2010). Flavonoids: new roles for old molecules. Journal of Intigrative Plant Biology 52, 98–111. doi: 10.1111/j.1744-7909.2010.00905.x 20074144

[B9] Cannon S. B. Mitra A. Baumgarten A. Young N. D. May G. J. (2004). The roles of segmental and tandem gene duplication in the evolution of large gene families in arabidopsis thaliana. BMC Plant Biology 4, 1–21. doi: 10.1186/1471-2229-4-10 15171794 PMC446195

[B10] Chapman M. A. Leebens-Mack J. H. Burke J. M. (2008). Positive selection and expression divergence following gene duplication in the sunflower CYCLOIDEA gene family. Mol. Biol. Evol. 25, 1260–1273. doi: 10.1093/molbev/msn001 18390478

[B11] Chen C. Chen H. Zhang Y. Thomas H. R. Frank M. H. He Y.. (2020). TBtools: an integrative toolkit developed for interactive analyses of big biological data. Molecular Plant 13, 1194–1202. doi: 10.1016/j.molp.2020.06.009 32585190

[B12] Chen H. Yang Q. Chen K. Zhao S. Zhang C. Pan R.. (2019). Integrated microRNA and transcriptome profiling reveals a miRNA-mediated regulatory network of embryo abortion under calcium deficiency in peanut (Arachis hypogaea l.). BMC Genomics 20, 1–17. doi: 10.1186/s12864-019-5770-6 31113378 PMC6528327

[B13] Dai X. Zhuang Z. Zhao P. X. (2018). psRNATarget: A plant small RNA target analysis server, (2017 release). Nucleic Acids Res. 46, W49–W54. doi: 10.1093/nar/gky316 29718424 PMC6030838

[B14] Davin L. B. Lewis N. G. (1992). “Phenylpropanoid metabolism: Biosynthesis of monolignols, lignans and neolignans, lignins and suberins,” in Phenolic metabolism in plants (New York: Springer), 325–375.

[B15] Donald R. Cashmore A. R. (1990). Mutation of either G box or I box sequences profoundly affects expression from the arabidopsis rbcS-1A promoter. The EMBO Journal 9, 1717–1726. doi: 10.1002/j.1460-2075.1990.tb08295.x 2347304 PMC551874

[B16] Dudareva N. Pichersky E. J. (2008). Metabolic engineering of plant volatiles. Current Opinion in Biotechnology 19, 181–189. doi: 10.1016/j.copbio.2008.02.011 18394878

[B17] Gao Y. Li J. Strickland E. Hua S. Zhao H. Chen Z.. (2004). An arabidopsis promoter microarray and its initial usage in the identification of HY5 binding targets *in vitro* . Plant Molecular Biology 54, 683–699. doi: 10.1023/B:PLAN.0000040898.86788.59 15356388

[B18] Gasteiger E. Hoogland C. Gattiker A. Wilkins M. R. Appel R. D. Bairoch A. J. (2005). Protein identification and analysis tools on the ExPASy server. 571–607. doi: 10.1385/1-59259-890-0:571 10027275

[B19] Giordano D. Provenzano S. Ferrandino A. Vitali M. Pagliarani C. Roman F.. (2016). Characterization of a multifunctional caffeoyl-CoA O-methyltransferase activated in grape berries upon drought stress. Plant Physiol. Biochem. 101, 23–32. doi: 10.1016/j.plaphy.2016.01.015 26851572

[B20] Giuliano G. Pichersky E. Malik V. Timko M. Scolnik P. Cashmore A. (1988). An evolutionarily conserved protein binding sequence upstream of a plant light-regulated gene. PNAS 85, 7089–7093. doi: 10.1073/pnas.85.19.7089 2902624 PMC282129

[B21] Gonzales M. D. Archuleta E. Farmer A. Gajendran K. Grant D. Shoemaker R.. (2005). The legume information system (LIS): an integrated information resource for comparative legume biology. Nucleic Acids Res. 33, D660–D665. doi: 10.1093/nar/gki128 15608283 PMC540082

[B22] Goujon T. Sibout R. Pollet B. Maba B. Nussaume L. Bechtold N.. (2003). A new arabidopsis thaliana mutant deficient in the expression of O-methyltransferase impacts lignins and sinapoyl esters. Plant Molecular Biology 51, 973–989. doi: 10.1023/A:1023022825098 12777055

[B23] Guo D. Chen F. Inoue K. Blount J. W. Dixon R. A. (2001). Downregulation of caffeic acid 3-o-methyltransferase and caffeoyl CoA 3-o-methyltransferase in transgenic alfalfa: impacts on lignin structure and implications for the biosynthesis of G and s lignin. The Plant Cell 13, 73–88. doi: 10.1105/tpc.13.1.73 11158530 PMC102215

[B24] Hamberger B. Ellis M. Friedmann M. de Azevedo Souza C. Barbazuk B. Douglas C. (2007). Genome-wide analyses of phenylpropanoid-related genes in populus trichocarpa, arabidopsis thaliana, and oryza sativa: The populus lignin toolbox and conservation and diversification of angiosperm gene families. Canadian Journal of Botany 85, 1182–1201. doi: 10.1139/B07-098

[B25] Hu D. Liu X. Wang C. Yang H. Li H. Ruan R.. (2015). Expression analysis of key enzyme genes in lignin synthesis of culm among different lodging resistances of common buckwheat (Fagopyrum esculentum moench.). Sci. Agric. Sin. 48, 1864–1872. doi: 10.3864/j.issn.0578-1752.2015.09.20

[B26] Huerta-Cepas J. Szklarczyk D. Heller D. Hernández-Plaza A. Forslund S. K. Cook H.. (2019). eggNOG 5.0: A hierarchical, functionally and phylogenetically annotated orthology resource based on 5090 organisms and 2502 viruses. Nucleic Acids Research 47, D309–D314. doi: 10.1093/nar/gky1085 30418610 PMC6324079

[B27] Jeffares D. C. Penkett C. J. Bähler J. (2008). Rapidly regulated genes are intron poor. Trends in Genetics 24, 375–378. doi: 10.1016/j.tig.2008.05.006 18586348

[B28] Joshi C. P. Chiang V. L. (1998). Conserved sequence motifs in plant s-adenosyl-L-methionine-dependent methyltransferases. Plant Molecular Biology 37, 663–674. doi: 10.1023/A:1006035210889 9687070

[B29] Kim J. Choi B. Cho B.-K. Lim H.-S. Kim J. B. Natarajan S.. (2013). Molecular cloning, characterization and expression of the caffeic acid O-methyltransferase (COMT) ortholog from kenaf (Hibiscus cannabinus). Plant Omics 6, 246–253.

[B30] Kolkman J. A. Stemmer W. P. (2001). Directed evolution of proteins by exon shuffling. Nat. Biotechnol. 19, 423–428. doi: 10.1038/88084 11329010

[B31] Kota P. Guo D. Zubieta C. Noel J. Dixon R. A. (2004). O-Methylation of benzaldehyde derivatives by “lignin specific” caffeic acid 3-o-methyltransferase. Phytochemistry 65, 837–846. doi: 10.1016/j.phytochem.2004.01.017 15081283

[B32] Kumar S. Stecher G. Li M. Knyaz C. Tamura K. J. (2018). MEGA X: Molecular evolutionary genetics analysis across computing platforms. Molecular Biology and Evolution 35, 1547. doi: 10.1093/molbev/msy096 29722887 PMC5967553

[B33] Lamesch P. Berardini T. Z. Li D. Swarbreck D. Wilks C. Sasidharan R.. (2012). The arabidopsis information resource (TAIR): improved gene annotation and new tools. Nucleic Acids Res. 40, D1202–D1210. doi: 10.1093/nar/gkr1090 22140109 PMC3245047

[B34] Lavin M. Herendeen P. S. Wojciechowski M. F. (2005). Evolutionary rates analysis of leguminosae implicates a rapid diversification of lineages during the tertiary. Systematic Biology 54, 575–594. doi: 10.1080/10635150590947131 16085576

[B35] Lescot M. Déhais P. Thijs G. Marchal K. Moreau Y. Van de Peer Y.. (2002). PlantCARE, a database of plant cis-acting regulatory elements and a portal to tools for in silico analysis of promoter sequences. Nucleic Acids Res. 30, 325–327. doi: 10.1093/nar/30.1.325 11752327 PMC99092

[B36] Li W. Lu J. Lu K. Yuan J. Huang J. Du H.. (2016). Cloning and phylogenetic analysis of brassica napus l. caffeic acid O-methyltransferase 1 gene family and its expression pattern under drought stress. PloS One 11, e0165975. doi: 10.1007/s11103-006-0029-4 27832102 PMC5104432

[B37] Li H. M. Rotter D. Hartman T. G. Pak F. E. Havkin-Frenkel D. Belanger F. C. (2006). Evolution of novel O-methyltransferases from the vanilla planifolia caffeic acid O-methyltransferase. Plant Molecular Biology 61, 537–552. doi: 10.1584/jpestics.31.47 16830185

[B38] Li J.-J. Zhang G. Yu J.-h. Li Y.-y. Huang X.-h. Wang W.-J.. (2015). Molecular cloning and characterization of caffeic acid 3-o-methyltransferase from the rhizome of ligusticum chuanxiong. Biotechnology Letters 37, 2295–2302. doi: 10.1007/s10529-015-1917-y 26254784

[B39] Lin F. Yamano G. Hasegawa M. Anzai H. Kawasaki S. Kodama O. (2006). Cloning and functional analysis of caffeic acid 3-o-methyltransferase from rice (Oryza sativa). Journal of Pesticide Science 31, 47–53. doi: 10.1584/jpestics.31.47

[B40] Lin S.-J. Yang Y.-Z. Teng R.-M. Liu H. Li H. Zhuang J. (2021). Identification and expression analysis of caffeoyl-coenzyme a O-methyltransferase family genes related to lignin biosynthesis in tea plant (Camellia sinensis). Protoplasma 258, 115–127. doi: 10.1007/s00709-020-01555-4 32929631

[B41] LIU S.-j. HUANG Y.-h. HE C.-j. Cheng F. ZHANG Y.-w. (2016a). Cloning, bioinformatics and transcriptional analysis of caffeoyl-coenzyme a 3-o-methyltransferase in switchgrass under abiotic stress. Journal of Integrative Agriculture 15, 636–649. doi: 10.1016/S2095-3119(16)61363-1

[B42] Liu X. Luo Y. Wu H. Xi W. Yu J. Zhang Q.. (2016b). Systematic analysis of O-methyltransferase gene family and identification of potential members involved in the formation of O-methylated flavonoids in citrus. Gene 575, 458–472. doi: 10.1016/j.gene.2015.09.048 26407870

[B43] Liu Q. Luo L. Zheng L. (2018). Lignins: biosynthesis and biological functions in plants. International Journal of Molecular Sciences 19, 335. doi: 10.3390/ijms19020335 29364145 PMC5855557

[B44] Livak K. J. Schmittgen T. D. (2001). Analysis of relative gene expression data using real-time quantitative PCR and the 2– ΔΔCT method. Methods 25, 402–408. doi: 10.1006/meth.2001.1262 11846609

[B45] Ma Q.-H. (2009). The expression of caffeic acid 3-o-methyltransferase in two wheat genotypes differing in lodging resistance. Journal of Experimental Botany 60, 2763–2771. doi: 10.1093/jxb/erp132 19451187 PMC2692018

[B46] Ma Q.-H. Luo H.-R. (2015). Biochemical characterization of caffeoyl coenzyme a 3-o-methyltransferase from wheat. Planta 242, 113–122. doi: 10.1007/s00425-015-2295-3 25854602

[B47] Moura J. C. M. S. Bonine C. A. V. de Oliveira Fernandes Viana J. Dornelas M. C. Mazzafera P. (2010). Abiotic and biotic stresses and changes in the lignin content and composition in plants. Journal of Integrative Plant Biology 52, 360–376. doi: 10.1111/j.1744-7909.2010.00892.x 20377698

[B48] Nguyen T.-N. Son S. Jordan M. C. Levin D. B. Ayele B. T. (2016). Lignin biosynthesis in wheat (Triticum aestivum l.): Its response to waterlogging and association with hormonal levels. BMC Plant Biol. 16, 1–16. doi: 10.1186/s12870-016-0717-4 26811086 PMC4727291

[B49] Peng D. Chen X. Yin Y. Lu K. Yang W. Tang Y.. (2014). Lodging resistance of winter wheat (Triticum aestivum l.): Lignin accumulation and its related enzymes activities due to the application of paclobutrazol or gibberellin acid. Field Crops Research 157, 1–7. doi: 10.1016/j.fcr.2013.11.015

[B50] Rakoczy M. Femiak I. Alejska M. Figlerowicz M. Podkowinski (2018). Sorghum CCoAOMT and CCoAOMT-like gene evolution, structure, expression and the role of conserved amino acids in protein activity. Mol. Genet. Genomics 293, 1077–1089. doi: 10.1007/s00438-018-1441-6 29721721 PMC6153501

[B51] Ralph J. Lundquist K. Brunow G. Lu F. Kim H. Schatz P. F.. (2004). Lignins: natural polymers from oxidative coupling of 4-hydroxyphenyl-propanoids. Phytochemistry Review 3, 29–60. doi: 10.1023/B:PHYT.0000047809.65444.a4

[B52] Raza A. Charagh S. Abbas S. Hassan M. U. Saeed F. Haider S.. (2023). Assessment of proline function in higher plants under extreme temperatures. Plant Biol. doi: 10.1111/plb.13510 36748909

[B53] Raza A. Charagh S. García-Caparrós P. Rahman M. A. Ogwugwa V. H. Saeed F.. (2022a). Melatonin-mediated temperature stress tolerance in plants. GM Crops Food 13, 196–217. doi: 10.1080/21645698.2022.2106111 35983948 PMC9397135

[B54] Raza A. Mubarik M. S. Sharif R. Habib M. Jabeen W. Zhang C.. (2022b). Developing drought-smart, ready-to-grow future crops. Plant Genome, e20279. doi: 10.1002/tpg2.20279 36366733 PMC12807413

[B55] Raza A. Su W. Gao A. Mehmood S. S. Hussain M. A. Nie W.. (2021a). Catalase (CAT) gene family in rapeseed (Brassica napus l.): Genome-wide analysis, identification, and expression pattern in response to multiple hormones and abiotic stress conditions. Int. J. Mol. Sci. 22, 4281. doi: 10.3390/ijms22084281 33924156 PMC8074368

[B56] Raza A. Tabassum J. Kudapa H. Varshney R. K. (2021b). Can omics deliver temperature resilient ready-to-grow crops? Crit. Rev. Biotechnol. 41, 1209–1232. doi: 10.1080/07388551.2021.1898332 33827346

[B57] Roje S. (2006). S-Adenosyl-L-methionine: beyond the universal methyl group donor. Phytochemistry 67, 1686–1698. doi: 10.1016/j.phytochem.2006.04.019 16766004

[B58] Savard O. T. Bertrand D. El-Mabrouk N. (2011). “Evolution of orthologous tandemly arrayed gene clusters,” in BMC bioinformatics, 12, 1–12. (Galway, Ireland: BioMed Central) 10.1186/1471-2105-12-S9-S2 PMC3283317 22152029

[B59] Sharif Y. Chen H. Deng Y. Ali N. Khan S. Zhang C.. (2022). Cloning and functional characterization of a pericarp abundant expression promoter (AhGLP17-1P) from peanut (Arachis hypogaea l.). Front. Genet. 12. doi: 10.3389/fgene.2021.821281 PMC8811503 35126474

[B60] Song S. Zhou H. Sheng S. Cao M. Li Y. Pang X. (2017). Genome-wide organization and expression profiling of the SBP-box gene family in Chinese jujube (Ziziphus jujuba mill.). 18, 1734. doi: 10.3390/ijms18081734 PMC5578124 28809790

[B61] Struck A. W. Thompson M. L. Wong L. S. Micklefield J. (2012). S-adenosyl-methionine-dependent methyltransferases: highly versatile enzymes in biocatalysis, biosynthesis and other biotechnological applications. ChemBioChem 13, 2642–2655. doi: 10.1002/cbic.201200556 23180741

[B62] Szklarczyk D. Gable A. L. Lyon D. Junge A. Wyder S. Huerta-Cepas J.. (2019). STRING v11: Protein–protein association networks with increased coverage, supporting functional discovery in genome-wide experimental datasets. Nucleic Acids Res. 47, D607–D613. doi: 10.1093/nar/gky1131 30476243 PMC6323986

[B63] Wang C. Wang X. Li J. Guan J. Tan Z. Zhang Z.. (2022). Genome-wide identification and transcript analysis reveal potential roles of oligopeptide transporter genes in iron deficiency induced cadmium accumulation in peanut. Front. Plant Sci. 13. doi: 10.3389/fpls.2022.894848 PMC9131082 35646039

[B64] Wang Y. Wang X. Paterson A. H. (2012). Genome and gene duplications and gene expression divergence: a view from plants. Ann. New York Acad. Sci. 1256, 1–14. doi: 10.1111/j.1749-6632.2011.06384.x 22257007

[B65] Wang D. Zhang Y. Zhang Z. Zhu J. Yu J. (2010). KaKs_Calculator 2.0: a toolkit incorporating gamma-series methods and sliding window strategies. Genomics, Proteomics & Bioinformatics 8, 77–80. doi: 10.1016/S1672-0229(10)60008-3 PMC5054116 20451164

[B66] Wani S. H. Kumar V. Khare T. Tripathi P. Shah T. Ramakrishna C.. (2020). miRNA applications for engineering abiotic stress tolerance in plants. Biologia 75, 1063–1081. doi: 10.2478/s11756-019-00397-7

[B67] Xu L. Dong Z. Fang L. Luo Y. Wei Z. Guo H.. (2019). OrthoVenn2: a web server for whole-genome comparison and annotation of orthologous clusters across multiple species. Nucleic Acids Research 47, W52–W58. doi: 10.1093/nar/gkz333 31053848 PMC6602458

[B68] Yang Z. Bielawski J. (2000). Statistical methods for detecting molecular adaptation. Trends in Ecology and Evolution 15, 496–503. doi: 10.1016/S0169-5347(00)01994-7 11114436 PMC7134603

[B69] Yaqoob H. Tariq A. Bhat B. A. Bhat K. A. Nehvi I. B. Raza A.. (2023). Integrating genomics and genome editing for orphan crop improvement: a bridge between orphan crops and modern agriculture system. GM Crops Food 14, 1–20. doi: 10.1080/21645698.2022.2146952 PMC9828793 36606637

[B70] Ye Z.-H. Kneusel R. E. Matern U. Varner J. (1994). An alternative methylation pathway in lignin biosynthesis in zinnia. The Plant Cell 6, 1427–1439. doi: 10.1105/tpc.6.10.1427 7994176 PMC160531

[B71] Yoshihara N. Fukuchi-Mizutani M. Okuhara H. Tanaka Y. Yabuya T. (2008). Molecular cloning and characterization of O-methyltransferases from the flower buds of iris hollandica. Journal of Plant Physiology 165, 415–422. doi: 10.1016/j.jplph.2006.12.002 17383769

[B72] Yu C. S. Chen Y. C. Lu C. H. Hwang J. K. (2006). Prediction of protein subcellular localization. Proteins: Structure, Function, and Bioinformatics 64, 643–651. doi: 10.1002/prot.21018 16752418

[B73] Yuan Z. Fang Y. Zhang T. Fei Z. Han F. Liu C.. (2018). The pomegranate (Punica granatum l.) genome provides insights into fruit quality and ovule developmental biology. Plant Biotechnology Journal 16, 1363–1374. doi: 10.1111/pbi.12875 29271050 PMC5999313

[B74] Zhang X. Chen B. Wang L. Ali S. Guo Y. Liu J.. (2021). Genome-wide identification and characterization of caffeic acid O-methyltransferase gene family in soybean. Plants 10, 2816. doi: 10.3390/plants10122816 34961287 PMC8703356

[B75] Zhang J. Li Y. Jia H.-X. Li J.-B. Huang J. Lu M.-Z.. (2015). The heat shock factor gene family in salix suchowensis: a genome-wide survey and expression profiling during development and abiotic stresses. Frontiers in Plant Science 6, 748. doi: 10.3389/fpls.2015.00748 26442061 PMC4584977

[B76] Zhou J.-M. Seo Y. W. Ibrahim R. K. (2009). Biochemical characterization of a putative wheat caffeic acid O-methyltransferase. Plant Physiology and Biochemistry 47, 322–326. doi: 10.1016/j.plaphy.2008.11.011 19211254

[B77] Zhuang W. Chen H. Yang M. Wang J. Pandey M. K. Zhang C.. (2019). The genome of cultivated peanut provides insight into legume karyotypes, polyploid evolution and crop domestication. Nature Genetics 51, 865–876. doi: 10.1038/s41588-019-0402-2 31043757 PMC7188672

