# Supplementary material for: In-silico identification and characterization of O-methyltransferase gene family in peanut (Arachis hypogaea L.) reveals their putative roles in development and stress tolerance
Source: Front Plant Sci. 2023 Mar 31;14:1145624. doi: 10.3389/fpls.2023.1145624 (PMC10102615; doi:10.3389/fpls.2023.1145624)
Supplement: Supplementary file 1 [file DataSheet_1.zip › Supplementary Materials/Supplementary Figures.docx]

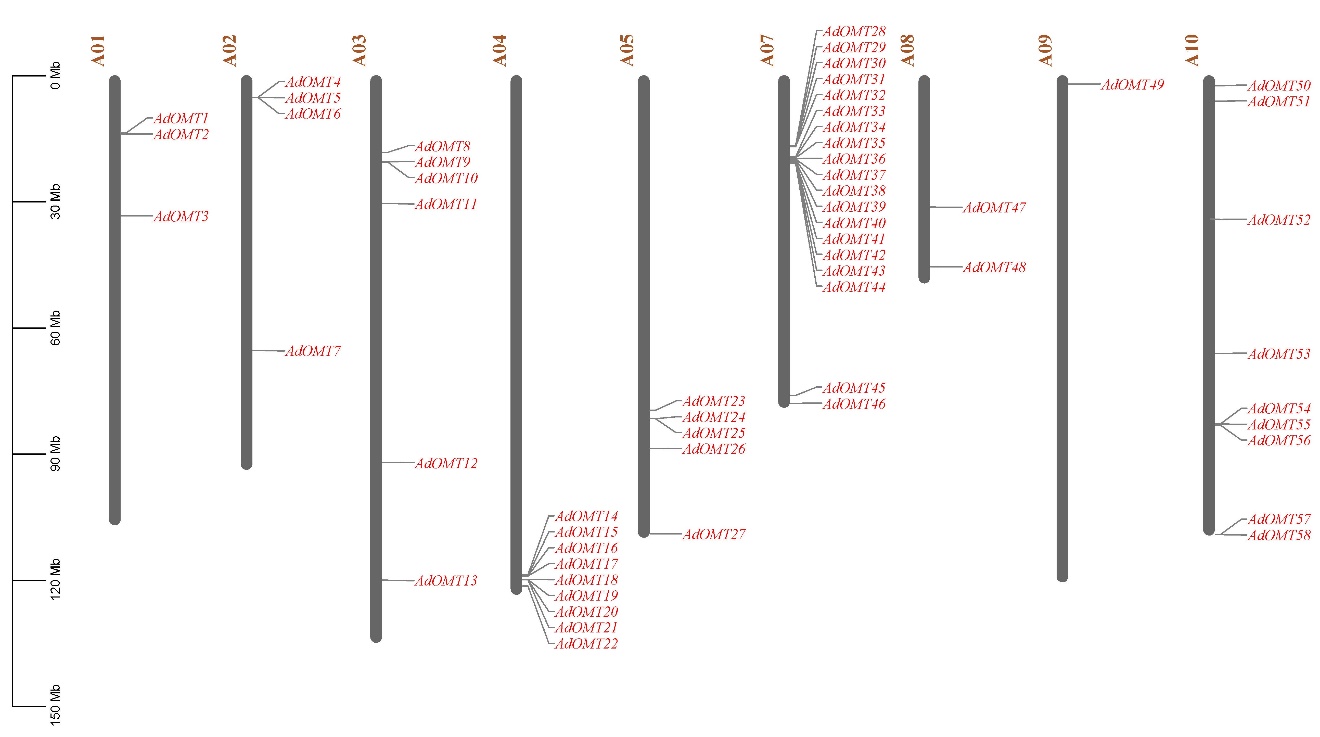


Supplementary Figure 1. Chromosomal distribution of Arachis duranensis O-Methyltransferase genes (AdOMTs). Chromosome A09 possessed only one *AdOMT* gene, while the highest number of genes is 19, present on chromosome A07.


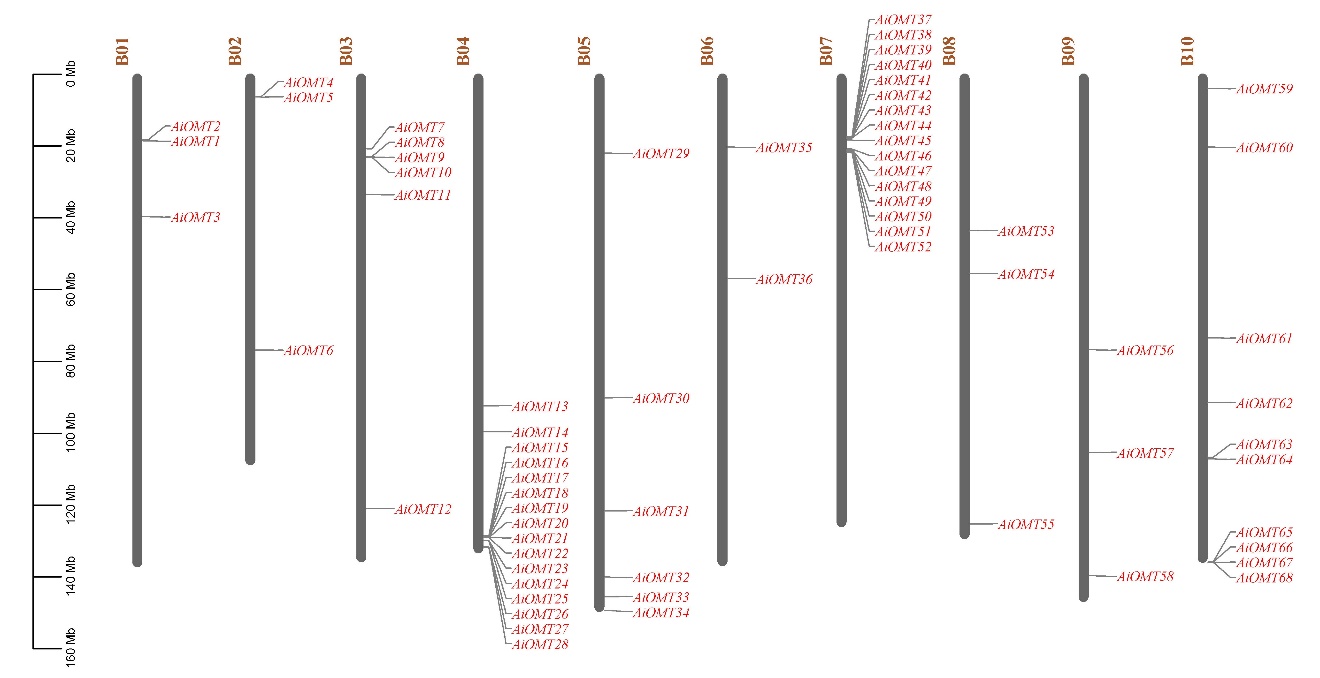


Supplementary Figure 2. Chromosomal distribution of Arachis ipaensis O-Methyltransferase genes (AiOMTs). Chromosomes B06 had the least number of *AiOMTs* (two), while chromosomes B04 and B07 possessed the highest number of *AiOMTs* (16 genes each).


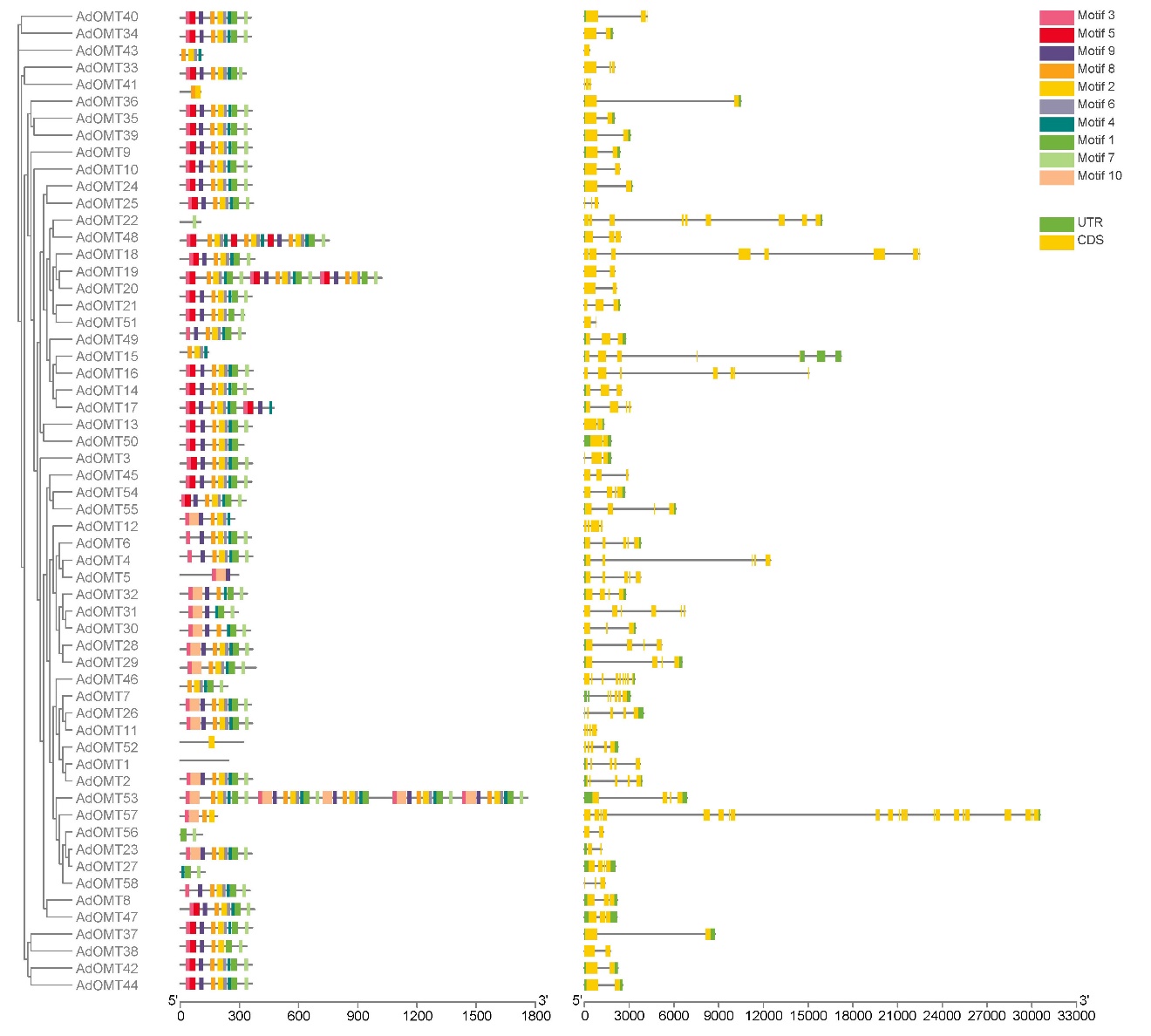


Supplementary Figure 3. Conserved motifs distribution patterns and gene structure (exons-introns distribution) of *Arachis duranensis* OMT (*AdOMTs*) genes.


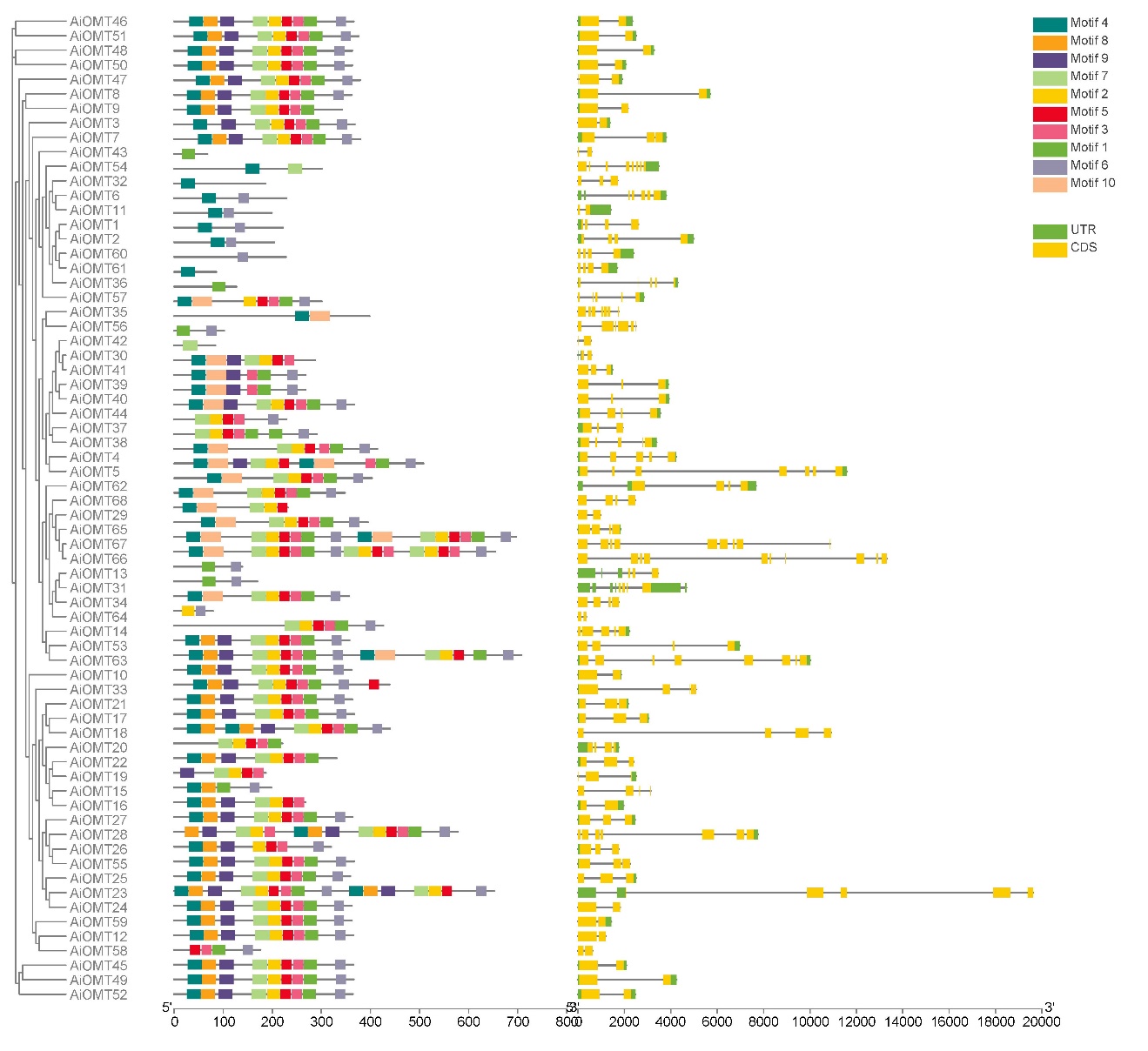


Supplementary Figure 4. Conserved motifs distribution patterns and gene structure (exons-introns distribution) of *Arachis ipaensis* OMT (*AiOMTs*) genes.


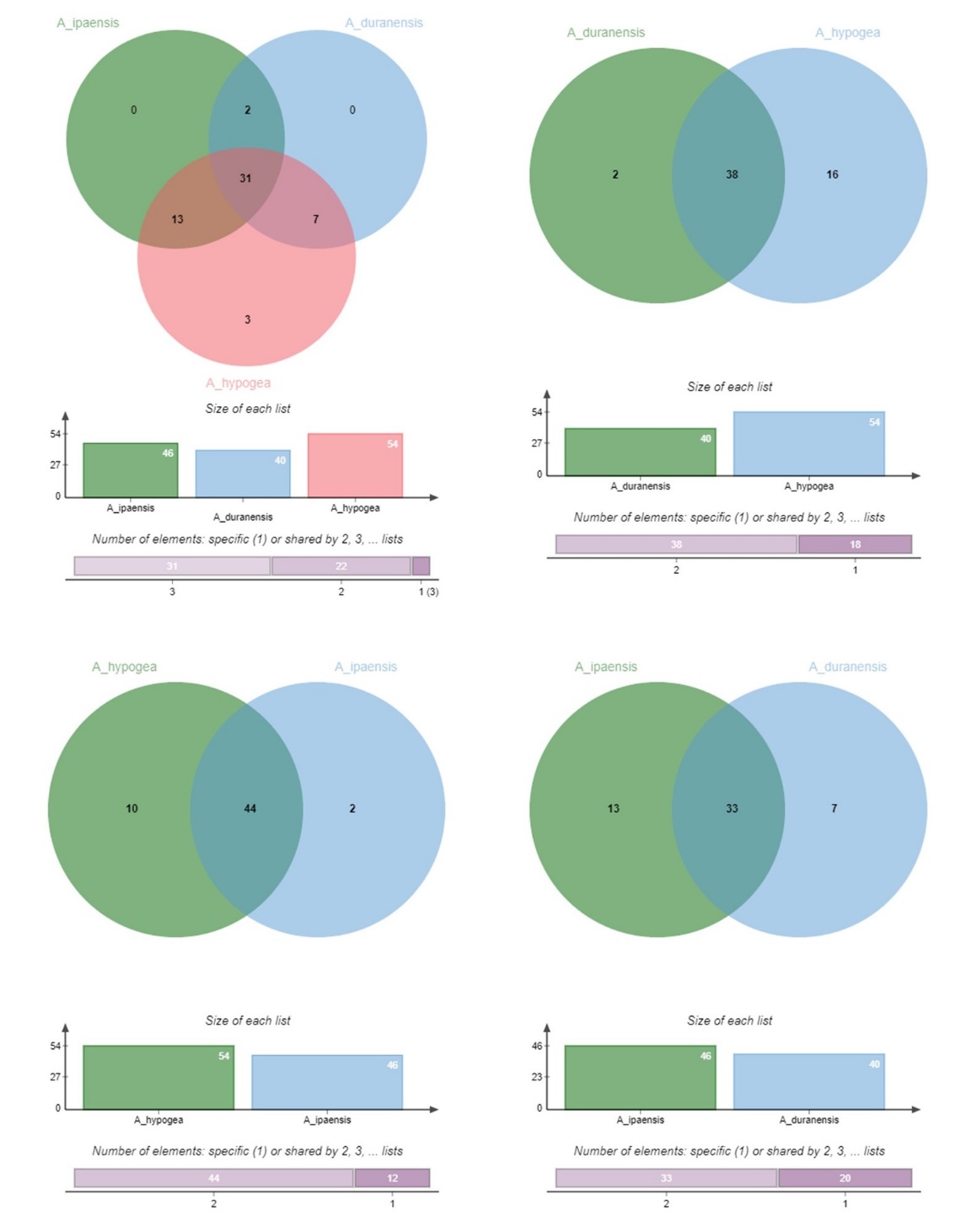


Supplementary Figure 5. Orthologous genes’ clusters among diploid and tetraploid species of peanut. (a) orthologous gene clusters among *A. hypogea*, *A. duranensis*, and *A. ipaensis* (b) orthologous gene clusters among *A. hypogea* and *A. duranensis* (c) orthologous gene clusters among *A. duranensis* and *A. ipaensis* (d) orthologous gene clusters among *A. hypogaea* and *A. ipaensis*.
